# Targeting Glutamine Metabolism Transporter SLC25A22 Enhances CD8+ T‐Cell Function and Anti‐PD‐1 Therapy Efficacy in Cervical Squamous Cell Carcinoma: Integrated Metabolomics, Transcriptomics and T‐Cell‐Incorporated Tumor Organoid Studies

**DOI:** 10.1002/advs.202502225

**Published:** 2025-06-27

**Authors:** Tingting Ren, Junjun Qiu, Fanghua Chen, Qian Jiang, Qinqin Liu, Tong Wu, Hua Jiang, Keqin Hua

**Affiliations:** ^1^ Department of Gynecology Obstetrics and Gynecology Hospital of Fudan University 419 Fangxie Road Shanghai 200011 China; ^2^ Shanghai Key Laboratory of Female Reproductive Endocrine‐Related Diseases 413 Zhaozhou Road Shanghai 200011 China

**Keywords:** anti‐PD‐1 therapy, Cervical squamous cell carcinoma, glutamine metabolism, metabolic profiling, transcriptional analysis

## Abstract

Cervical squamous cell carcinoma(CSCC) represents formidable challenge in clinical oncology, exacerbated by poor prognosis and resistance to current treatments, including anti‐PD‐1 therapy, highlighting the urgent need for alternative therapeuties. Metabolic characteristics have emerged as potential drivers of treatment resistance and immune evasion. Herein, 1) based on metabolomic and transcriptomic analyses of 44 CSCC and 18 normal tissues, glutamine‐enriched and immunosuppressive microenvironment is identified in CSCC. 2) Integrative metabolomic and transcriptomic analyses revealed the glutamine metabolism transporter SLC25A22 as a key mediator in high glutamine metabolism, immune checkpoint activation and CD8+T‐cell cytotoxicity. 3) Immunohistochemistry(IHC), multiplex IHC, and flow cytometry validation with clinical CSCC samples revealed not only increased SLC25A22, PD‐1 expression and reduced CD8+T‐cell cytotoxicity in CSCC but also increased SLC25A22 expression in high PD‐L1 expressed CSCC patients, suggesting the potential of targeting SLC25A22 for enhancing CD8+T‐cell cytotoxicity and improving anti‐PD‐1 efficacy, especially in high PD‐L1 expressed patients. 4) Novelly, 3D‐CSCC organoids are constructed, replicating parental tumor features, and 3D‐T‐cell‐incorporated CSCC organoid models, replicating the interaction between tumor cells and CD8+T cells, for in vitro experiments. 5) Importantly, it is validated through in vitro 3D T‐cell‐incorporated CSCC organoid models and in vivo animal experiments that targeting the glutamine metabolism transporter SLC25A22, showed promise in enhancing CD8+T‐cell cytotoxicity and sensitizing anti‐PD‐1 therapy. These findings provided insights for future clinical trials exploring metabolic modulation to improve immunotherapy responses in CSCC patients.

## Graphical abstract

Through metabolomic and transcriptomic analyses, a glutamine‐enriched, immunosuppressive microenvironment is identified in cervical squamous cell carcinoma (CSCC), highlighting the glutamine transporter SLC25A22 as a key regulator of glutamine metabolism, immune checkpoint activation, and CD8+ T‐cell cytotoxicity. Targeting SLC25A22 in vitro and in vivo enhanced the immune response and sensitized tumors to anti‐PD‐1 therapy, suggesting potential for improving CSCC immunotherapy outcomes.

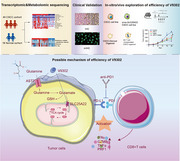

## 1 Introduction

Cervical cancer (CC) is a prevalent gynecologic disease and ranks fourth among female malignant tumors in terms of incidence and mortality.^[^ ^1^, ^2^ ^]^ Among its subtypes, cervical squamous cell carcinoma (CSCC) is the most common, accounting for 70% of cases.^[^ ^3^ ^]^ The standard treatments for CC are surgery, chemotherapy, and radiation therapy, but their efficacy remains relatively low.^[^ ^4^, ^5^ ^]^ For instance, during chemoradiation therapy with cisplatin, up to 40% of CC patients experience disease recurrence.^[^ ^6^ ^]^ Recently, immunotherapies utilizing immune checkpoint inhibitors, particularly those targeting programmed cell death protein 1 (PD‐1) and its ligand (PD‐L1), have revolutionized cancer treatment.^[^ ^7^ ^]^ However, the clinical response to PD‐1/PD‐L1 blockade remains limited, with a clinical trial showing an objective response rate of only 16.4% in CSCC patients.^[^ ^8^ ^]^ Thus, novel strategies are urgently needed to improve immunotherapy efficacy in CSCC.

The tumor microenvironment (TME) is instrumental in tumor progression and immune escape, directly impacting immunotherapy outcomes.^[^ ^9^ ^]^ Emerging evidence suggests that tumor‐infiltrating immune cells, such as CD8+ T cells and macrophages, play a key role in immune surveillance and are linked to immunotherapy responses in CSCC.^[^ ^10^, ^11^ ^]^ However, tumor cells can evade immune surveillance by exploiting negative checkpoint regulatory pathways, such as the PD‐1 pathway, leading to the dysfunction of cytotoxic T lymphocytes.^[^ ^12^ ^]^ Additionally, tumors create a metabolically hostile environment by depleting nutrients and accumulating metabolic byproducts such as lactate or glutamine.^[^ ^13^ ^]^ This hostile environment directly impairs immune cell function and diminishes the cancer immunotherapy efficacy.^[^ ^14^, ^15^ ^]^ Hence, investigating not only the characteristics of the TME but also metabolic alterations, particularly glutamine metabolism, is crucial for optimizing immunotherapy strategies.

Glutamine metabolism is essential for tumor growth by providing cancer cells with the necessary nutrients for their survival and proliferation.^[^ ^16^ ^]^ Although glutamine is a nonessential amino acid, tumors import large amounts of glutamine from the TME, which limits its availability to infiltrating T cells and inhibits their activation.^[^ ^17^ ^]^ Recent studies suggest that inhibiting glutamine transport—for example, using the glutamine transporter inhibitor V‐9302—enhances CD8+ T‐cell activity and improves immunotherapy responses in triple‐negative breast cancer.^[^ ^18^, ^19^ ^]^ Furthermore, combining glutamine inhibition with immunotherapy has shown promise in remodeling the TME in lung and colon cancers^[^ ^13^, ^20^ ^]^ However, while glutamine metabolism has been implicated in radiotherapy resistance in cervical cancer, its role in immunotherapy remains largely unexplored.^[^ ^21^ ^]^ In addition, glutamine metabolism distinctly affects both tumor cells and immune cells within the tumor immune microenvironment (TIME).^[^ ^22^ ^]^ Therefore, there is an urgent need to explore the specific impact of glutamine metabolism on the TIME and the potential value of targeting glutamine metabolism to sensitize CSCC patients to immunotherapy.

In the present study, we aimed to explore the metabolic and immune profiles of CSCC by conducting metabolomic and transcriptomic analyses of 44 CSCC samples and 18 normal samples, and the analysis revealed the presence of a glutamine‐enriched, immunosuppressive microenvironment in CSCC, which has significant implications. By integrating metabolomic and transcriptomic analyses, we further discovered that the glutamine transporter SLC25A22 might act as a vital mediator that links glutamine metabolism, immune checkpoint activation, and the cytotoxicity of CD8+ T cells in CSCC. Moreover, through validation of CSCC clinical samples using IHC, mIHC, and flow cytometry, we not only revealed a concomitant increase in SLC25A22 and PD‐1 expression and reduced CD8+ T‐cell cytotoxicity but also identified increased SLC25A22 levels in CSCC patients with elevated PD‐L1 expression, suggesting the potential value of targeting SLC25A22 for enhancing CD8+ T‐cell cytotoxicity and improving anti‐PD‐1 therapy efficacy, especially in patients with high PD‐L1 expression. Furthermore, to explore the therapeutic value of targeting SLC25A22 in CSCC, we initially developed innovative 3D CSCC organoids that closely resemble the morphology and genomic characteristics of CSCC tumors. Additionally, we created 3D T‐cell‐incorporated CSCC organoid models to mimic the interaction between tumor cells and T cells. Using this novel in vitro model, we demonstrated that targeting the glutamine transporter SLC25A22 with V‐9302 enhanced CD8+ T‐cell cytotoxicity and improved the effectiveness of anti‐PD‐1 therapy. Finally, our in vivo xenograft model experiments supported the efficacy of targeting SLC25A22 with V‐9302 in improving CD8+ T‐cell cytotoxicity and the response to anti‐PD‐1 therapy. These findings not only elucidate the unique metabolic features and immune microenvironment in CSCC but also suggest that anti‐PD‐1 therapy sensitivity can be increased by targeting the glutamine transporter SLC25A22, which could lay the foundation for future clinical research.

## 2 Results

### 2.1 Metabolomic and Transcriptomic Sequencing Revealed that CSCC Exhibits High Glutamine Related Metabolism and an Immunosuppressive Microenvironment

Given that tumor metabolism can significantly influence immune cell activation and function, thereby impairing immune cell function and promoting cancer immunotherapy efficacy, we conducted metabolomic and transcriptomic sequencing to comprehensively analyze the metabolic and immune landscapes of 44 CSCC tissue samples and 18 normal tissue samples. The patients’ clinical characteristics are shown in Table S1 (Supporting Information).

#### 2.1.1 Metabolomic Sequencing: CSCC Exhibits High Glutamine Related Metabolism

**Figure 1:**
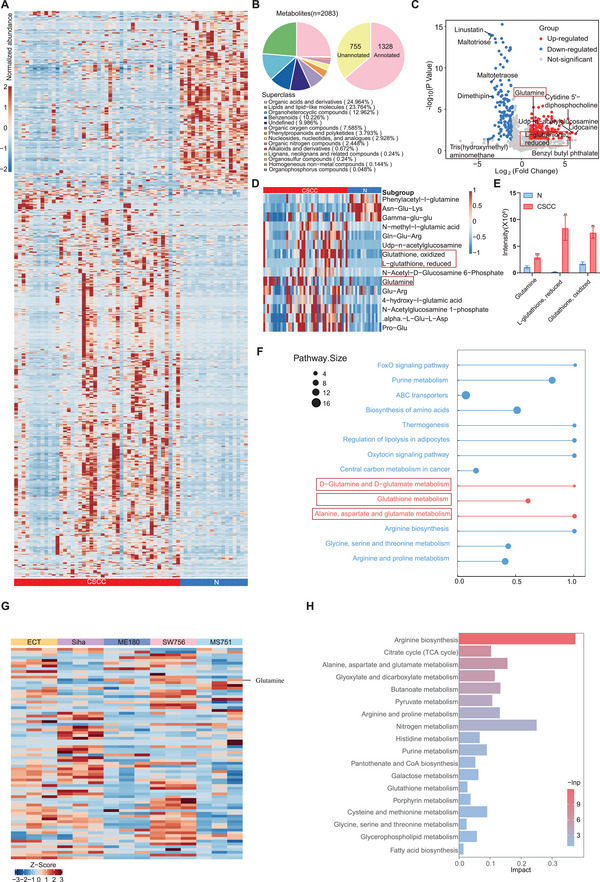
CSCC exhibits high glutamine metabolism microenvironment: A) Unsupervised hierarchical clustering reveals distinct metabolic profiles in CSCC patients. B) Pie chart showed types and proportions of 2083 identified metabolites (Left) and the numbers and proportions of annotated metabolites (Right) in our study. C) A volcano plot showed differentially expressed metabolites between CSCC and normal tissue (*p* value<0.05, Foldchange>1.5). Top 10 significantly differentially expressed metabolites and glutamine‐related metabolites were labeled in the volcano plot. D) A heatmap showed glutamine‐related metabolites in CSCC and normal tissues. E) A bar plot showed higher intensity of glutamine and glutathione (reduced and oxidized form) in CSCC compared to normal tissues. (*t*‐test, ***p < 0.001, **p < 0.01) F) A pathway‐based analysis of metabolomic changes between CSCC and normal tissues. The DA score captures the average, gross changes for all metabolites in a pathway. A score of 1 indicates that all measured metabolites in the pathway increase in the tumor compared to normal tissues. Pathways with no less than three measured metabolites were used for DA score calculation. G) A heatmap of differentially expressed metabolites among CSCC cell lines (Siha, ME180, SW756, MS751) and cervical normal cell line (ECT). H) A KEGG analysis revealed top 18 accumulated pathways of differentially expressed metabolites between CSCC cell lines and ECT cell. Abbreviations: CSCC, cervical squamous cell cancer; DA, differential abundance; KEGG, Kyoto Encyclopedia of Genes and Genomes.

We detected a total of 2083 metabolites from 44 CSCC and 18 normal tissues, among which 1328 were annotated via the Kyoto Encyclopedia of Genes and Genomes (KEGG) database or the Human Metabolome Database (HMDB) (**Figure** **1**B). To characterize the distinct metabolic profile of CSCC patients, we performed unsupervised hierarchical clustering based on the 1328 annotated metabolites from 44 CSCC and 18 normal samples (Figure 1A). A total of 118 differentially expressed metabolites (DEMs) (*P* < 0.05) were identified between CSCC tissues and normal tissues. Among these dysregulated DEMs, glutamine‐related metabolites, including glutamine (Gln) and glutathione (GSH) were significantly enriched in CSCC (Figure 1C–E). Additionally, KEGG pathway‐based differential abundance (DA) analysis demonstrated that most DEMs were associated with amino acid metabolism pathways, particularly those related to glutamine metabolism (Figure 1F). Furthermore, to validate the presence of elevated glutamine metabolism in CSCC, we employed metabolomics analysis of CSCC cell lines (Siha/ME180/SW756/MS751) and normal cervical cell line (ECT). Notably, most CSCC cell lines exhibited higher levels of glutamine‐related metabolites compared to ECT cells (Figure 1G; Figure S1A, Supporting Information). DA analysis further confirmed that the DEMs between the CSCC and ECT cell lines were significantly accumulated in the glutamine metabolism‐related pathway, underscoring the critical role of elevated levels of glutamine‐related metabolites in the CSCC (Figure 1H). Overall, these metabolic profiling results revealed that CSCC tissues exhibited higher levels of glutamine‐related metabolism than normal tissues.

#### 2.1.2 Transcriptomic Sequencing: CSCC Exhibits an Immunosuppressive Microenvironment

**Figure 2:**
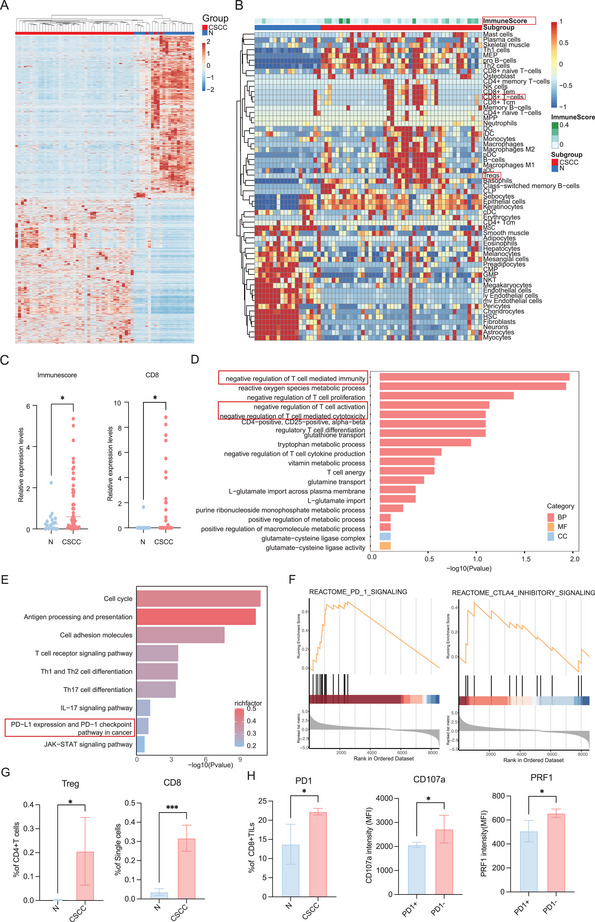
CSCC exhibits immunosuppressive microenvironment: A) Unsupervised hierarchical clustering reveals distinct transcriptomic profiles in CSCC patients. B) A heatmap of immune cell inflitration and immune score in CSCC and normal tissues based on RNA‐sequencing data. C) A boxplot showed relative expression levels of immunescore, CD8+T cell. (*t*‐test, *p < 0.05, ***p < 0.001,****p < 0.0001) D) GO analysis showed the identified DEGs were enriched negative regulation of T cell‐mediated immunity. E) KEGG analysis showed the identified DEGs were enriched in PD‐L1 expression and PD‐1 checkpoint pathway in cancer. F) GSEA analysis indicated that DEGs mapped to PD‐1 signaling and CTLA‐4 inhibitory signaling. G) Flow cytometry analysis revealed the potential higher infiltration of Treg and CD8+T cells in CSCC, H) elevated PD‐1 expression on CD8+ T cells and lower cyctotoxic effectors(CD107a and PRF1) in negative PD‐1 expression CD8+T cells. (*t*‐test, *p < 0.05, ***p < 0.001) Abbreviations: CSCC, cervical squamous cell cancer; GO, Gene Ontology; KEGG, Kyoto Encyclopedia of Genes and Genomes; DEG, Differentially expressed genes; GSEA, gene set enrichment; PRF1, perforin 1.

The heatmap (**Figure** **2**A) illustrates the differentially expressed genes (DEGs) between the CSCC and normal samples, as identified by transcriptomic sequencing. Notably, CSCC patients exhibited increased immune cell infiltration (Figure 2B). Specifically, we observed not only a higher abundance of immunosuppressive cells but also increased infiltration of immunoreactive CD8+ T‐cell infiltration (Figure 2C). To decipher the biological and regulatory functions that these immune cells within the TIME, we conducted Gene Ontology (GO) and KEGG enrichment analyses. Notably, GO analysis of the DEGs revealed enrichment in the negative regulation of T‐cell‐mediated immunity (Figure 2D). Similarly, KEGG pathway analysis revealed that the DEGs were associated with PD‐L1 expression and the PD‐1 checkpoint pathway in cancer, contributing to an immunosuppressive milieu (Figure 2E). Moreover, gene set enrichment analysis (GSEA) corroborated these findings, indicating that the DEGs were highly mapped to PD‐1 signaling pathways (Figure 2F), which may underlie the dysfunction of infiltrated CD8+ T cells and the immunosuppressive microenvironment. To further validate these observations, flow cytometry analysis confirmed the potential increased infiltration of immune cells, such as Tregs and CD8+ T cells (Figure 2G). Additionally, we observed elevated PD‐1 expression on CD8+ T cells in CSCC; and decreased cytotoxic effector expression in CD8+ T cells with low PD‐1 expression (Figure 2H). These findings suggest that CD8+ T cells function is likely hindered by the PD‐1 checkpoint pathway. In summary, our results indicate that CSCC harbors an immunosuppressive microenvironment, potentially driven by the infiltration of immunosuppressive cells and the inhibition of CD8+ T‐cell function through the PD‐1 checkpoint pathway.

### 2.2 Integrative Metabolomic and Transcriptomic Analysis Revealed the Importance of the Glutamine Metabolism Transporter SLC25A22 in the Immunosuppressive Microenvironment in CSCC

**Figure 3:**
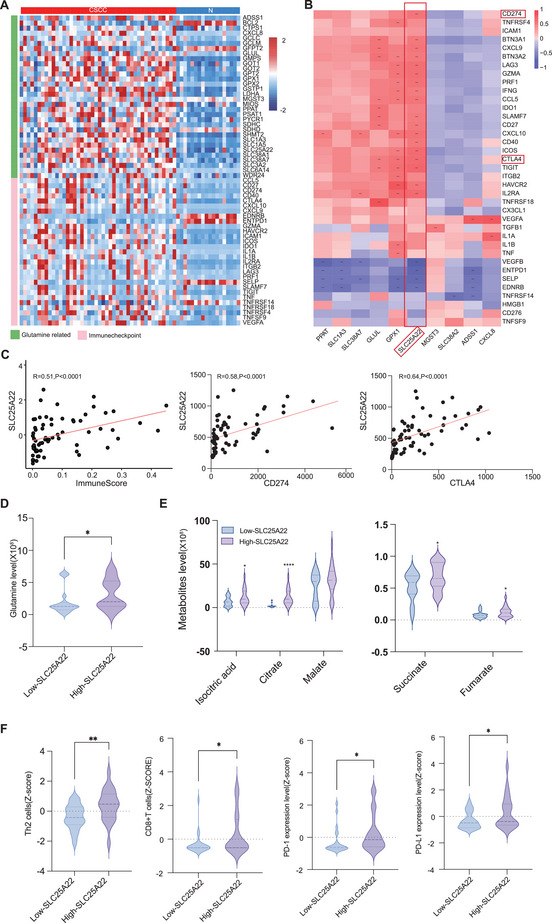
Glutamine metabolism transporter SLC25A22 served as a key player connecting glutamine metabolism, immune checkpoint activation, and CD8+ T cell cytotoxicity in CSCC. A) A heatmap demonstrated the expression levels of glutamine related genes and immune checkpoint related genes. B) The correlation heatmap illustrated the pairwise correlation matrix of glutamine related and immunecheckpoint gene expression levels in CSCC. SLC25A22 was identified with extensive and strong relationship with immunecheckpoint genes such as PD‐1 and CTLA‐4 (Person's test,*p < 0.05, ***p < 0.001,****p < 0.0001). C) Scatter plot illustrating the correlation between SLC25A22 and immunescore, CD274 (PD‐L1) and CTLA‐4(Person's test). D) The violin plot showed higher levels of glutamine in high‐SLC25A22 compared to low‐SLC25A22 group (*t*‐test, *p < 0.05). E) The violin plot showed higher levels of isocitric acid, citrate, succinate and fumarate in high‐SLC25A22 compared to low‐SLC25A22 group (*t*‐test, Mann‐Whitey test,*p < 0.05,****p < 0.0001). F) The violin plot showed enhanced infiltration of Th2 cells, CD8+T cells, along with higher expression levels of PD‐1/PD‐L1 in high‐SLC25A22 expression group. (*t*‐test, *p < 0.05, **p < 0.01) Abbreviations: CSCC, cervical squamous cell cancer.

To thoroughly investigate the potential relationships between glutamine metabolism and the immunosuppressive microenvironment, we conducted an integrative analysis of metabolomics and transcriptomics data. Specifically, we assessed the expression levels of glutamine‐related genes and immunosuppressive regulatory genes in CSCC samples. A heatmap analysis (**Figure** **3**A) demonstrated the upregulation of not only glutamine‐related genes, including those encoding glutamine‐utilizing enzymes (GLS and GFPT1, etc.) and glutamine transporters (SLC1A5, SLC38A1, SLC38A2, SLC6A14, and SLC25A22, etc) but also immune checkpoint genes, which play crucial roles as negative immunomodulators in CSCC. Further correlation analysis between these glutamine‐related genes and immune checkpoint genes revealed that the glutamine transporter gene SLC25A22 exhibited strong positive correlations with multiple immune checkpoint genes, such as CD274 (PD‐L1) and CTLA‐4 (Figure 3B,C), suggesting its potential role in activating immune checkpoints to hinder CD8+T‐cell immune function. Additionally, SLC25A22 also exhibited a strong correlation with key cytotoxic markers like IFN‐gamma (IFNγ), TNF‐alpha (TNFα), and CD107a, indicating its possible involvement in regulating CD8+ T‐cell activity (Figure S1B, Supporting Information). Furthermore, to elucidate the impact of SLC25A22 expression, we stratified the 44 CSCC patients into a high‐ (n = 22) and a low‐SLC25A22 expression groups (n = 22) based on median expression levels. Notably, compared to the low‐SLC25A22 expression group, the high‐SLC25A22 expression group not only exhibited significantly higher glutamine levels (Figure 3D), but also an increase in the content of metabolites involved in the tricarboxylic acid cycle that glutamine participates in (Figure 3E), indicating a potential positive correlation between SLC25A22 expression and glutamine metabolism. Moreover, the high‐SLC25A22 expression group displayed enhanced immune cell infiltration, including both immunosuppressive Th2 cells and CD8+ T‐cells, alongside elevated expression of crucial immune checkpoint molecules, such as PD‐1 (Figure 3F), which further supports the idea that SLC25A22 might be related to the cytotoxicity of damaged CD8+ T cells through immune checkpoints. Together, these data suggested that the glutamine metabolism transporter SLC25A22 contributes to the development of an immunosuppressive microenvironment within tumors and serves as a key player connecting glutamine metabolism, immune checkpoint activation, and CD8+ T‐cell cytotoxicity in CSCC.

### 2.3 Clinical Validation Demonstrated a Concurrent Increase in the Expression of the Glutamine Metabolism Transporter SLC25A22 and PD‐1/PD‐L1, Accompanied by a Reduction in CD8+ T‐Cell Cytotoxicity in CSCC Patients

**Figure 4:**
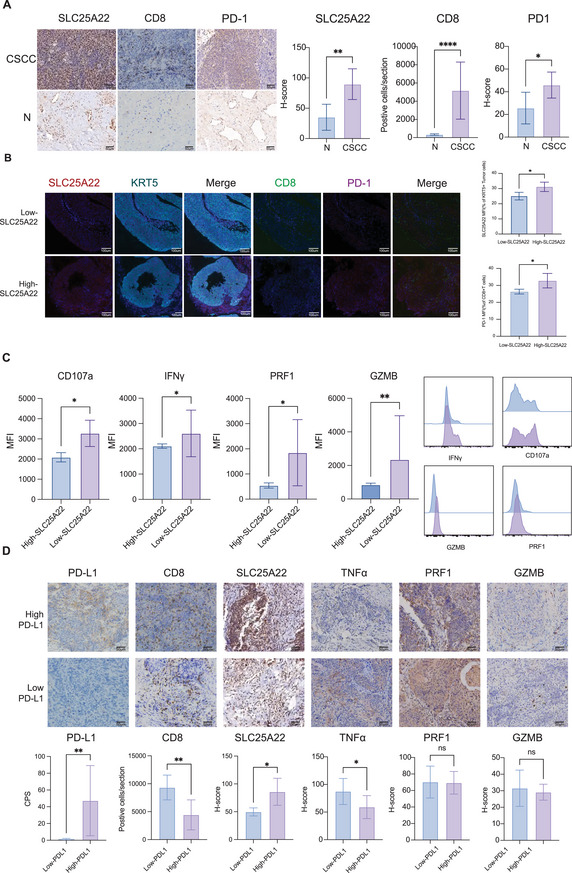
Clinical validation of expression levels of SLC25A22/PD1/PDL1/CD8/cytotoxic effectors in CSCC and normal tissues. A) Representative immunohistochemistry images of clinical specimen showing the expression levels of SLC25A22, CD8, and PD‐1 in CSCC and normal tissues. Original magnifications: ×100, scare bar:100 µm. B) Dual staining of serial sections showed expression level of SLC25A22 (red) on tumor cells (KRT5+) (green) and expression level of PD1 (red) on CD8+T cells (CD8+) (red).Original magnifications: ×200, scare bar:100µm(Left). Quantification of the SLC25A22+KRT5+tumor cells and PD‐1+CD8+T cells ratio per field for 4 CSCC specimens(Right). Three fields of each slide were randomly picked. C) The bar plot illustrated the MFI (Mean Fluorescence Intensity) values for CD107a, IFNγ, PRF1, and GZMB in high/low SLC25A22 group. The corresponding histogram overlays dipicting the expression of CD107a, IFNγ, PRF1, and GZMB were shown on the right. D) Representative immunohistochemistry images of clinical specimen showing the expression levels of PD‐L1, SLC25A22, CD8, PRF1 and GZMB in 20 CSCC. Original magnifications: ×100. Quantification of PD‐L1 CPS, number of CD8 positive cells and H‐score of PRF1 and GZMB of 20 CSCC specimens were shown below. Three fields of each slide were randomly picked. Experiments were performed independently for each specimen. *p* values were obtained by the Friedman test (A, B) and Mann‐Whitney U test (C, D). *p < 0.05, **p < 0.01, ***p < 0.001. Abbreviations: CSCC, cervical squamous cell cancer; IFNγ, interferon gamma; PRF1, perforin 1; GZMB, granzyme B; CPS, combined positive score; MFI, mean fluorescence intensity.

To further verify the importance of the glutamine transporter SLC25A22 in the immunosuppressive microenvironment of CSCC, we evaluated the expression levels of SLC25A22, the immune checkpoint PD‐1 and CD8, as well as the cytotoxicity of effector CD8+ T cells, in 20 CSCC samples and 3 normal samples with complete clinical and pathological information obtained from the original 44 cancer samples and 18 normal samples (Table S1, Supporting Information). Our analysis revealed that, compared with normal tissues, CSCC tissues exhibited higher expression of the glutamine transporter SLC25A22 and PD‐1, along with increased infiltration of CD8+ T cells (**Figure** **4**A). Subsequent mIHC further confirmed that SLC25A22 was predominantly localized on epithelial cells rather than CD8+T cells (Figure S2A, Supporting Information). Moreover, mIHC staining of four CSCC samples demonstrated that CSCC patients not only had elevated SLC25A22 expression but also elevated PD‐1 expression on CD8+ T cells, indicating a potential positive association between SLC25A22 on epithelial cells and PD‐1 expression on CD8+ T cells (Figure 4B). To determine whether high SLC25A22 expression is linked to CD8+ T‐cell cytotoxicity in CSCC, we conducted flow cytometry analysis. The results showed that CD107a, IFNY, GZMB, and PRF1—key markers of CD8+ T‐cell cytotoxic activity—were expressed at lower levels in the high‐SLC25A22 expression CSCC group than in the low‐SLC25A22 expression group (Figure 4C), suggesting that elevated SLC25A22 expression was also associated with reduced in CD8+ T‐cell cytotoxicity in CSCC.

Given the simultaneous increase in SLC25A22 and PD‐1 expression in CSCC patients, and considering that PD‐L1 expression serves as a predictive biomarker for immunotherapy response,^[^ ^22^ ^]^ we further analyzed the relationship between SLC25A22 and PD‐L1 expression. Based on the combined positive score (CPS) cutoff of 3, we classified the 20 CSCC patients into a high PD‐L1 expression group (n = 17) and a low PD‐L1 expression group (n = 3). Notably, patients in the high PD‐L1 expression group exhibited significantly higher SLC25A22 expression and lower CD8+ T‐cell cytotoxicity than those in the low PD‐L1 expression group (Figure 4D).

Inspired by the above exciting clinical findings of a concurrent increase in SLC25A22 and PD‐1 expression accompanied by a reduction in CD8+ T‐cell cytotoxicity in patients with CSCC, as well as the enrichment of SLC25A22 in the high PD‐L1 expression CSCC patients, we proposed that targeting the glutamine metabolism transporter SLC25A22 might have the potential to enhance CD8+ T‐cell cytotoxicity and reduce PD‐1 expression, thereby improving the efficacy of anti‐PD‐1 therapy, especially for high PD‐L1 expression CSCC patients. To verify this hypothesis, we next explored the therapeutic value of targeting the glutamine metabolism transporter SLC25A22 in in vitro and in vivo models.

### 2.4 Targeting the Glutamine Metabolism Transporter SLC25A22 with V‐9302 Enhanced the Cytotoxicity of CD8+ T Cells and the Efficacy of Anti‐PD‐1 Therapy in 3D T‐Cell‐Incorporated CSCC Organoids and Animal Experiments

#### 2.4.1 The Glutamine Metabolism Transporter SLC25A22 Played a Crucial Role in the Malignant Behavior of CSCC

**Figure 5:**
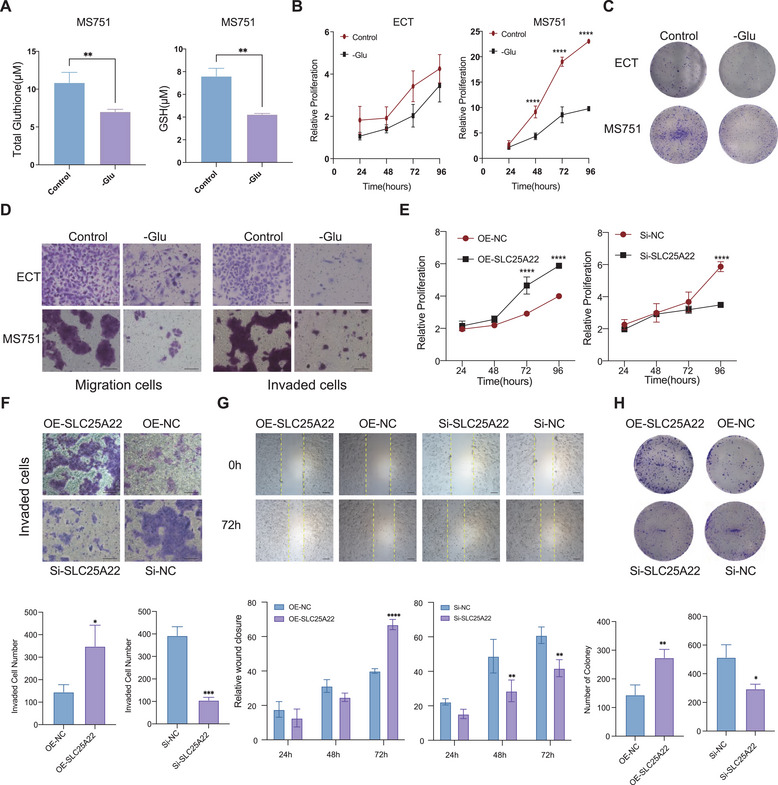
Role of glutamine metabolism and SLC25A22 in the malignant progression of CSCC. A) A bar plot showed lower level of glutamine and glutathione under glutamine‐deprived conditions in MS751 cell lines. (*t*‐test, **p < 0.01) B) CCK‐8 assay showed a significant reduction MS751 cell proliferation ability under glutamine‐deprived conditions. (One‐way ANOVA test, ****p < 0.0001). C) Representive images of cell clone of MS751 cell lines and ECT cell lines. D) Representive images of trasnwell migration and invasion experiments of MS751 and ECT cell lines under glutamine‐deprived conditions. Original magnifications: ×200, scare bar:100 µm. E) CCK‐8 assay showed a significant reduction of cell proliferation ability in SLC25A22 knockdown (Si‐SLC25A22) MS751 cell line and increment of cell proliferation ability in SLC25A22 overexpression (OE‐SLC25A22) MS751 cell line. F) Representive images of trasnwell invasion experiments of Si‐SLC25A22/Si‐NC/OE‐SLC25A22/OE‐NC cell lines. Original magnifications: ×200, scare bar:100 µm. G) Wound healing assay of Si‐SLC25A22/Si‐NC/OE‐SLC25A22/OE‐NC cell lines. Original magnifications: ×100, scare bar:100 µm. H) Representive images of cell clone of Si‐SLC25A22/Si‐NC/OE‐SLC25A22/OE‐NC cell lines.

To explore the role of elevated glutamine metabolism in CSCC, we measured the levels of glutathione, both in clinical samples of CSCC and normal tissue (Figure S3A, Supporting Information), as well as in CSCC cell lines and normal cervical cell line (Figure S3B, Supporting Information). Additionally, we examined glutathione levels (**Figure** **5**A; Figure S3C, Supporting Information), as well as cell proliferation (Figure 5B; Figure S3D, Supporting Information), colony formation (Figure 5C; Figure S3E, Supporting Information), invasion and migration (Figure 5D; Figure S3F, Supporting Information) abilities under glutamine‐deprived conditions. Our findings revealed that glutamine plays a critical role in promoting the malignant biological behavior of CSCC.

As is well known, SLC25A22, as a mitochondrial glutamate transporter, plays a critical role in mitochondrial energy metabolism involving glutamine and in the production of GSH.^[^ ^23^ ^]^ Previous studies have shown that SLC25A22 contributes to tumorigenesis in colorectal cancer by modulating associated energy metabolism and signaling pathway.^[^ ^24^ ^]^ To investigate its role in the biological behavior of CSCC, we developed MS751 cell lines with either knockdown or overexpression of SLC25A22. (Figure S3G, Supporting Information). Our findings revealed that SLC25A22 knockdown markedly suppressed malignant phenotypes, including proliferation, invasion, migration and colony ability, whereas its overexpression exhibited the opposite effects (Figure 5E–H). These results underscore the pivotal role of SLC25A22 in driving the malignant progression of tumor cells.

#### 2.4.2 Impact of V9302 on SLC25A22‐Mediated Glutamine Metabolism in CSCC

To evaluate the therapeutic potential of targeting glutamine metabolism, specifically its mitochondrial transporter SLC25A22, we investigated the efficacy of V9302, a known glutamine transporter inhibitor, in CSCC cells. Metabolic flux analysis demonstrated that V9302 significantly impaired the SLC25A22‐mediated glutamine metabolism in TCA cycle (Figure S4A, Supporting Information), highlighting that V9302 not only decreased intracellular glutamine uptake but also inhibited the activity of the mitochondrial transporter SLC25A22, responsible for glutamate transport across the mitochondrial membrane. Given that SLC25A22 is predominantly expressed in epithelial cells, it may play a significant role in modulating the function of CD8+ T cells and immunotherapeutic response.^[^ ^25^ ^]^ We hypothesize that targeting SLC25A22 could modulate the cytotoxicity of CD8+ T cells by influencing the communication between tumor cells and T cells. However, current 2D cellular models fail to accurately capture the complex interplay between tumor cells and T cells within the TIME. Therefore, there is a pressing need to develop more sophisticated models that better these interactions in CSCC.

#### 2.4.3 CSCC‐Derived Organoids: Miniature Replicas of the Original Tumor Tissue

**Figure 6:**
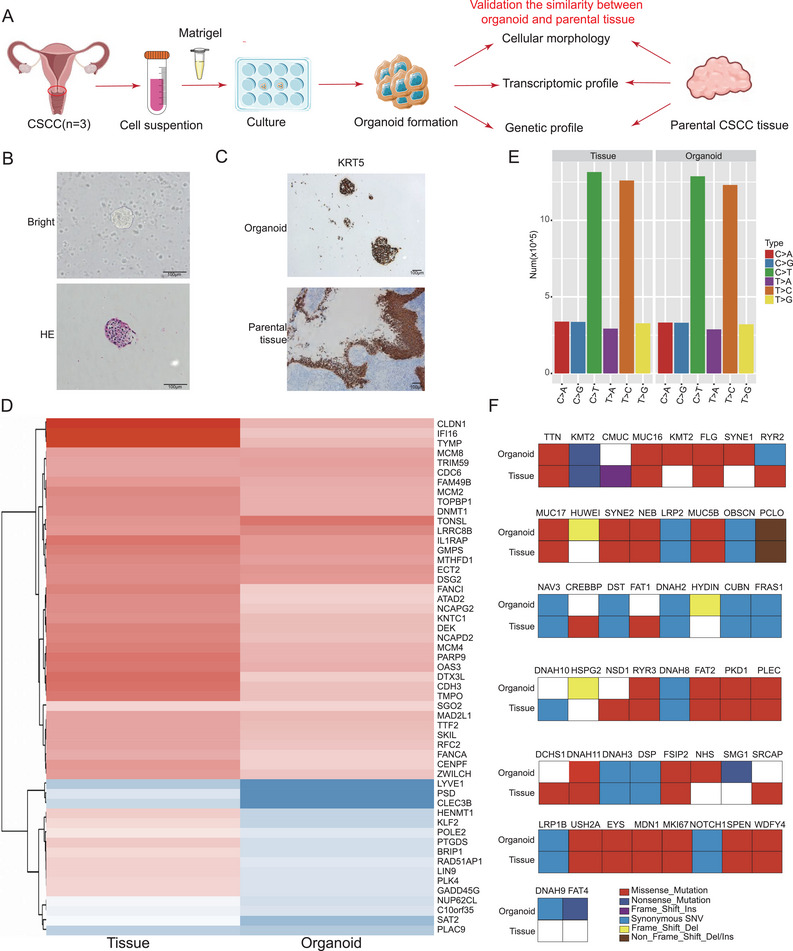
Construction of CSCC organoid and the similarity validation between organoid and parental tissue. A)Schematic diagram of building CSCC‐derived organoid. B) HE staining (Lower) and representative micrographs (Upper) showed the density structure in CSCC‐derived organoid. Original magnifications: ×200, scare bar: 100 µm. C) IHC staining showed biomarkers of CSCC tumor tissue were positively expressed in CSCC‐derived organoid (KRT5). Original magnifications: ×100, scare bar: 100 µm. D) Heatmap showed the similar gene expression profile between CSCC‐organoid and parental tissue. E) Bar graphs displayed the different contributions of SNV in CSCC‐derived organoid and original tumor tissue. F) The heatmap showed the top 50 gene mutation type in CSCC‐derived organoid and original tumor tissue. Abbreviations: CSCC, cervical squamous cell cancer; HE, hematoxylin‐eosin staining; IHC, immunohistochemistry; SNV, single‐nucleotide variants.

To recapitulate the interplay between tumor cells and T cells within the TIME of CSCC, we initially constructed CSCC‐derived organoids as a platform for in vitro emulation of tumor features (**Figure** **6**A). The H&E staining results demonstrated that the CSCC‐derived organoids retained the tissue architecture and cellular morphology of the original tumor tissue (Figure 6B). IHC staining confirmed that KRT5 expression, a marker of squamous cell differentiation, was preserved in the organoids (Figure 6C). Additionally, transcriptome sequencing of the organoids and their matched tissues revealed highly similar gene expression profiles, indicating that transcriptome features were largely preserved in the CSCC‐derived organoids (Figure 6D). Importantly, whole‐genome sequencing (WGS) analysis demonstrated a highly consistent mutation landscape between primary tumor tissue and CSCC‐derived organoids. We also observed the presence of frequently mutated genes (MUC16, MUC17, *KMT2*), further confirming the stable maintenance of the core mutation profile (Figure 6E,F). Collectively, these findings indicate that CSCC‐derived organoids faithfully recapitulate the histopathological and genetic characteristics of their parental tumors, providing a robust platform for studying tumor biology and potential therapeutic interventions.

#### 2.4.4 3D T‐Cell‐Incorporated CSCC Organoids: Recapitulating the Tumor Microenvironment Composed of CD8+ T Cells and Tumor Cells

**Figure 7:**
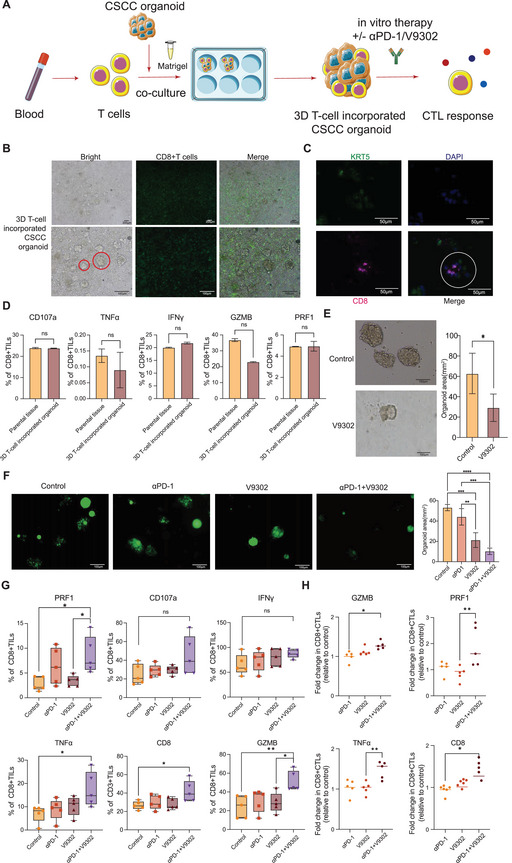
Construction of 3D T cell incorporated organoid and in vitro validation of V9302 targeting SLC25A22 as promising therapy to sensitize anti‐PD‐1 therapy: A) Schematic diagram of building 3D T‐cell incorporated CSCC organoid and in vitro experiments. B) Representative bright field images of 3D T‐cell incorporated CSCC organoid.Original magnifications: ×40 (Upper) x100 (Lower).Scare bar:200 µm (Upper) 100 µm (Lower). C) Representative IF images of 3D T‐cell incorporated CSCC organoid composed of KRT5+tumor cells (green), CD8+T cells (pink). Original magnifications: x100 (lower). Scare bar: 100 µm. D) Barplot showed the similar T cell cytotoxic effectors (CD107a, IFNγ, TNFa, PRF1, and GZMB) between 3D T‐cell incorporated CSCC organoid and parental tissues. E) Representative bright field images of CSCC organoid in control group and V9302 treatment group. Original magnifications: x100(lower). Scare bar: 100 µm. Quantification of CSCC organoid area was shown on the right. F) Representative IF images of 3D T‐cell incorporated CSCC organoid in control group and V9302 treatment group. Original magnifications: x100 (lower). Scare bar: 100 µm. Quantification of CSCC organoid area (green) was shown on the right. G) Percentages of cells expressing effector cytokines and cytolytic markers among CD45+CD8+ TILs from 3D T‐cell incorporated CSCC organoid treated by anti‐PD1 (αPD‐1), V9302, or combined anti‐PD1(αPD‐1)&V9302. (n = 5 for each group). Experiments were performed independently for each specimen. *p* values were obtained by the Friedman test (D, E, G), Wilcoxon matched‐pairs signed‐ranks test(F). *p < 0.05, **p < 0.01, ***p < 0.001, ns: not significant. Abbreviations: CSCC, cervical squamous cell cancer; IF, immunofluorescence; IFNγ, interferon gamma; PRF1, perforin 1; GZMB, granzyme B; TIL, tumor infiltrating lymphocytes.

To further explore the repercussions of glutamine metabolism on the dynamic interaction between tumor cells and CD8+ T cells, we constructed a 3D T‐cell‐incorporated CSCC organoid model (**Figure** **7**A), which was established by coculturing CSCC organoids with T cells isolated from PBMCs, embedding them within a Matrigel matrix to facilitate the formation of 3D T‐cell‐incorporated CSCC organoids. Morphological analysis revealed that we observed that T cells actively surrounded and infiltrated the CSCC organoids (Figure 7B). Additionally, mIHC staining confirmed the encapsulation of CD8+ T cells in the tumor organoids (Figure 7C). Flow cytometry analysis verified that CD8+ T lymphocytes were the predominant immune cell population in the co‐culture system (Figure S5A, Supporting Information), and functionally confirmed that the cytotoxic effectors secreted by T‐cell‐incorporated tumor organoids were similar to those in the original tumor(Figure 7D). In conclusion, our findings establish that CD8+ T cells encapsulated in tumor organoids recapitulate the interaction between T cells and tumor cells in CSCC in vitro and could serve as an effective and functional tool for studying T‐cell infiltration, cytotoxicity, and interactions with cancer cells in the TIME.

#### 2.4.5 Targeting the Glutamine Metabolism Transporter SLC25A22 with V‐9302 Enhanced the Cytotoxicity of CD8+ T Cells and the Efficacy of Anti‐PD‐1 Therapy in 3D T‐Cell‐Incorporated CSCC Organoids

Given our findings that increased SLC25A22 expression correlates with elevated PD‐1 expression and impaired CD8+ T cell cytotoxicity, we hypothesized that targeting the glutamine metabolism transporter SLC25A22 could enhance the cytotoxicity of CD8+ T cells and sensitize CSCC cells to PD‐1 therapy. To test this hypothesis, we conducted 3D in vitro validation experiments using the 3D T‐cell‐incorporated CSCC organoid model and evaluated the efficacy of V‐9302 in enhancing the efficacy of PD‐1. Encouragingly, the results demonstrated that V‐9302 not only inhibited tumor growth in CSCC organoids but also sensitized 3D T‐cell‐incorporated CSCC organoids to anti‐PD‐1 therapy (Figure 7E,F). Surprisingly, compared with anti‐PD‐1 monotherapy, anti‐PD‐1‐V‐9302 therapy increased the expression of effector cytokines (TNF‐α) and the release of cytolytic markers (CD107a, GZMB, and PRF1) in CD8+ T cells (Figure 7G).

Moreover, in the co‐culture system of CD8+ T cells with SLC25A22 knockdown/overexpression cell lines, we observed that knockdown of SLC25A22 led to an increase in the expression of key effector molecules in CD8+ T cells, including GZMB and CD107, whereas overexpression of SLC25A22 resulted in a reduction of these molecules (Figure S5B, Supporting Information). Furthermore, the combination of SLC25A22 knockdown with V9302 led to a more robust activation of CD8+ T cell effector functions (Figure S5C, Supporting Information). Flow cytometric analysis indicated that this enhanced immune response may be linked to a reduction in the expression of immune checkpoint markers on the surface of the cells, suggesting a potential mechanism for the observed synergistic effect (Figure S5D, Supporting Information).

These findings suggested that targeting the glutamine metabolism transporter SLC25A22 with V‐9302 increased CD8+ T‐cell cytotoxicity and the efficacy of anti‐PD‐1 therapy, which suggested that the use of V‐9302 in combination with anti‐PD‐1 therapy may be a promising treatment approach.

#### 2.4.6 Targeting the Glutamine Metabolism Transporter SLC25A22 with V‐9302 Enhanced the Cytotoxicity of CD8+ T Cells and the Therapeutic Activity of the Anti‐PD‐1 Agent In Vivo

**Figure 8:**
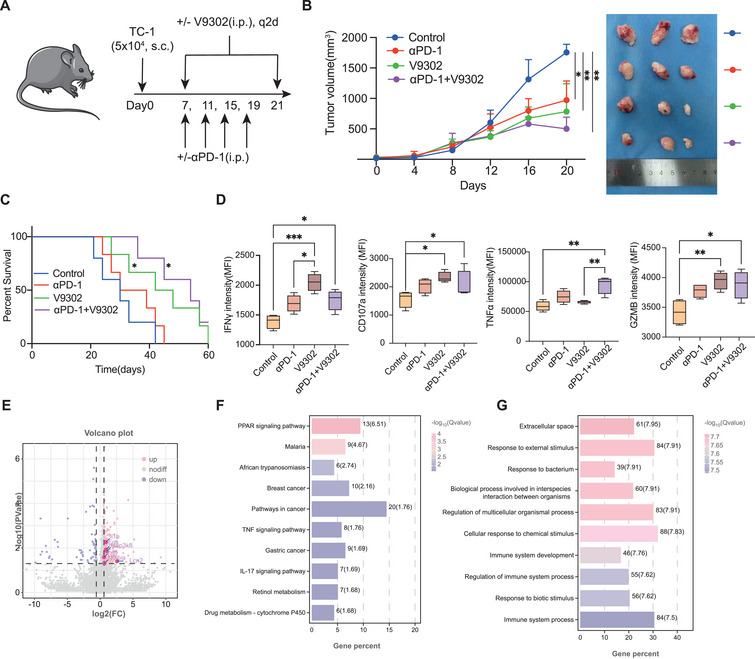
V‐9302 promotes antitumor efficiency of anti‐PD‐1 therapy in TC‐1 tumor‐bearing C57BL/6 mice. A) Schematic representation of the experimental strategy. C57BL/6 mice were given intraperitoneal injections of αPD‐1 on days 7, 11, 15, 19, and/or V9302 q2d after the injection of 5 × 10^4^ TC‐1 tumor cells subcutaneously on day 0 (n = 10 for each group). B)Tumor size in mice treated as described in A (n = 10 for each group) at various time points after the challenge (Left). Representative photos of tumors in each group on day 21 (Right). C)Survival of mice treated as described in A. The survival of the mice was determined by denoting the last day of ethical tumor size measurement as the time of sacrifice. D) Percentages of cells expressing effector cytokines and cytolytic markers among CD45+CD8+ TILs from in vivo model treated by anti‐PD1(αPD‐1), V9302 or combined anti‐PD1(αPD‐1)&V9302. (n = 10 for each group). Experiments were performed once for a total of 40 mice. E) A volcano plot showed DEGs between combined anti‐PD1(αPD‐1)&V9302 treatment and anti‐PD1 monotherapy. F)KEGG analysis showed the identified DEGs were enriched in TNF signaling and IL‐17 signaling pathway. G) GO analysis showed the identified DEGs were enriched in regulation of immune system process. *p* values were obtained by the Kruskal‐Wallis test (B‐C) and log‐rank test (D). *P<0.05, **P<0.01, ***P<0.001. Abbreviations: TIL, tumor infiltrating lymphocytes.

To further assess the therapeutic potential of the glutamine metabolism inhibitor V‐9302, we performed an in vivo study using the TC‐1 tumor‐bearing C57BL/6 mouse model, where initial tumor sizes were standardized at 50 mm^3^ across all groups (**Figure** **8**A). Intraperitoneal infusion of either anti‐PD‐1 (200 µg per mouse) or V‐9302 (400 µg per mouse) significantly reduced tumor growth, while the difference between the two treatment groups was not significant (Figure 8B). More encouragingly, combination treatment with V‐9302 and an anti‐PD‐1 antibody exhibited a superior effect in controlling tumor growth and prolonging the survival in tumor‐bearing mice compared to either monotherapy alone (Figure 8C). Flow cytometry analysis of tumor‐infiltrating CD8+ T cells revealed that cotreatment with V‐9302 and anti‐PD‐1 significantly enhanced the expression of cytolytic proteins (GZMB and PRF1) and effector cytokines (IFN‐γ and TNF‐α) compared to monotherapy groups. These results were consistent with our in vitro observations in the 3D T‐cell‐incorporated organoid model (Figure 8D).

To further explain the rationale for combining V9302 with immunotherapy to enhance its efficacy, we performed transcriptomic sequencing on mice from the control group, V9302 alone, PD‐1 alone, and the combination of V9302 and PD‐1. Compared to the PD‐1 monotherapy group, KEGG pathway enrichment analysis revealed significant enrichment in immune‐related pathways, such as the IL‐17 and TNF signaling pathways, in the combination therapy group (Figure 8E,F). GO analysis further highlighted the enrichment of the regulation of immune system process (Figure 8G). These findings suggest that V9302 may enhance the effectiveness of immunotherapy by modulating key immune‐related pathways, such as TNF and IL‐17 signaling pathway, thereby sensitizing the immune response.

Therefore, in these mouse models, the combination of the glutamine metabolism transporter inhibitor V‐9302 and anti‐PD‐1 therapy was more effective than was the monotherapy alone at promoting a sustained protective antitumor CD8+ T‐cell response and inhibiting the growth of implanted tumors in mice, further demonstrating that targeting the glutamine metabolism transporter SLC25A22 in tumor cells with V‐9302 can sensitize them to anti‐PD‐1 therapy through the dysregulation of immune‐related pathways.

**Figure 9:**
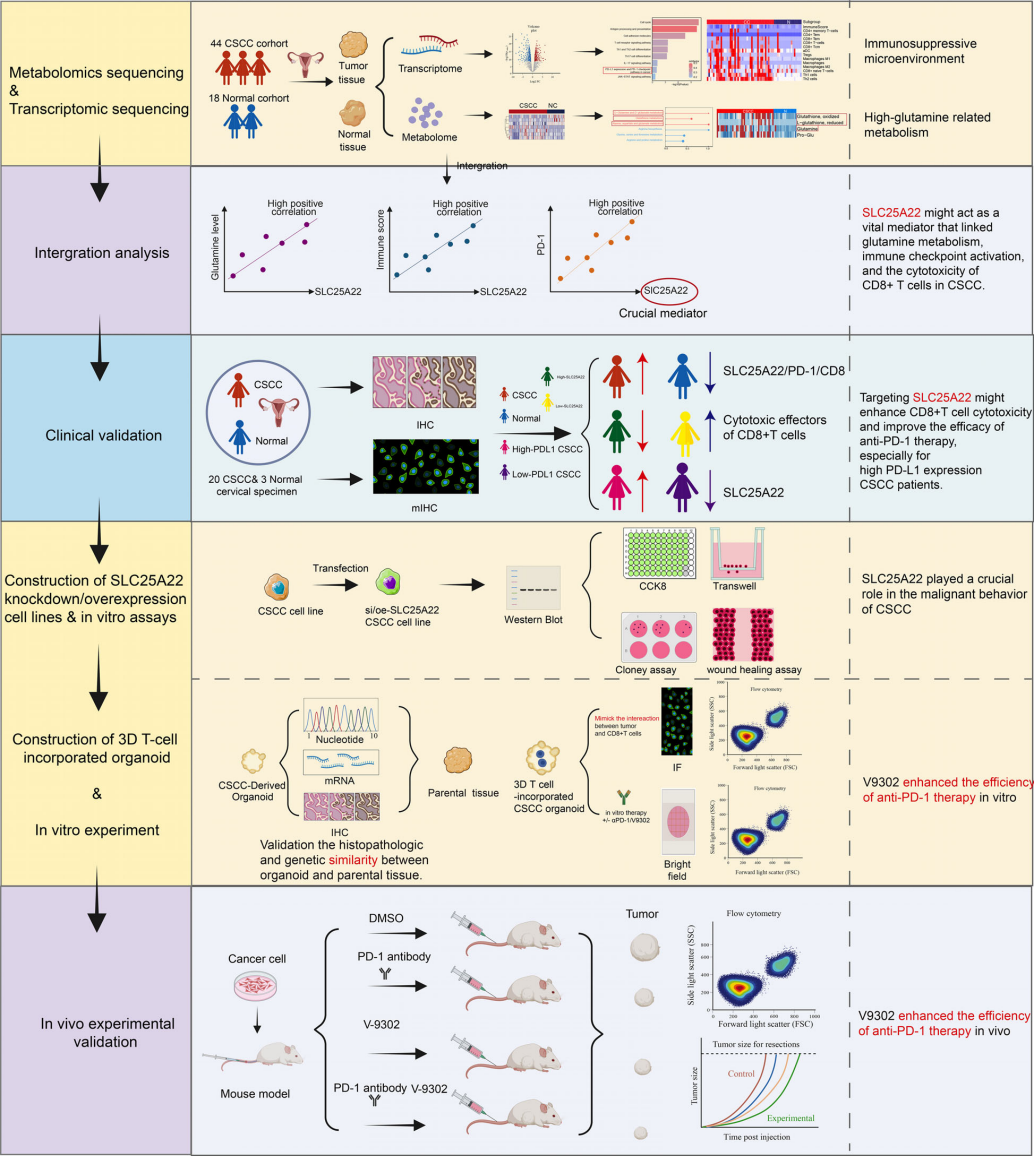
The overall flowchart and main conclusion of this experiment. Comprehensive flowchart illustrating the experimental workflow from initial setup to final analysis. The process involves metabolomics and transcriptomic sequencing, intergration analysis, construction of CSCC organoid, 3D T‐cell incorporated organoid, in vitro experiment and in vivo experimental validation. The main conclusion is presented on the right side of the figure.

**Figure 10:**
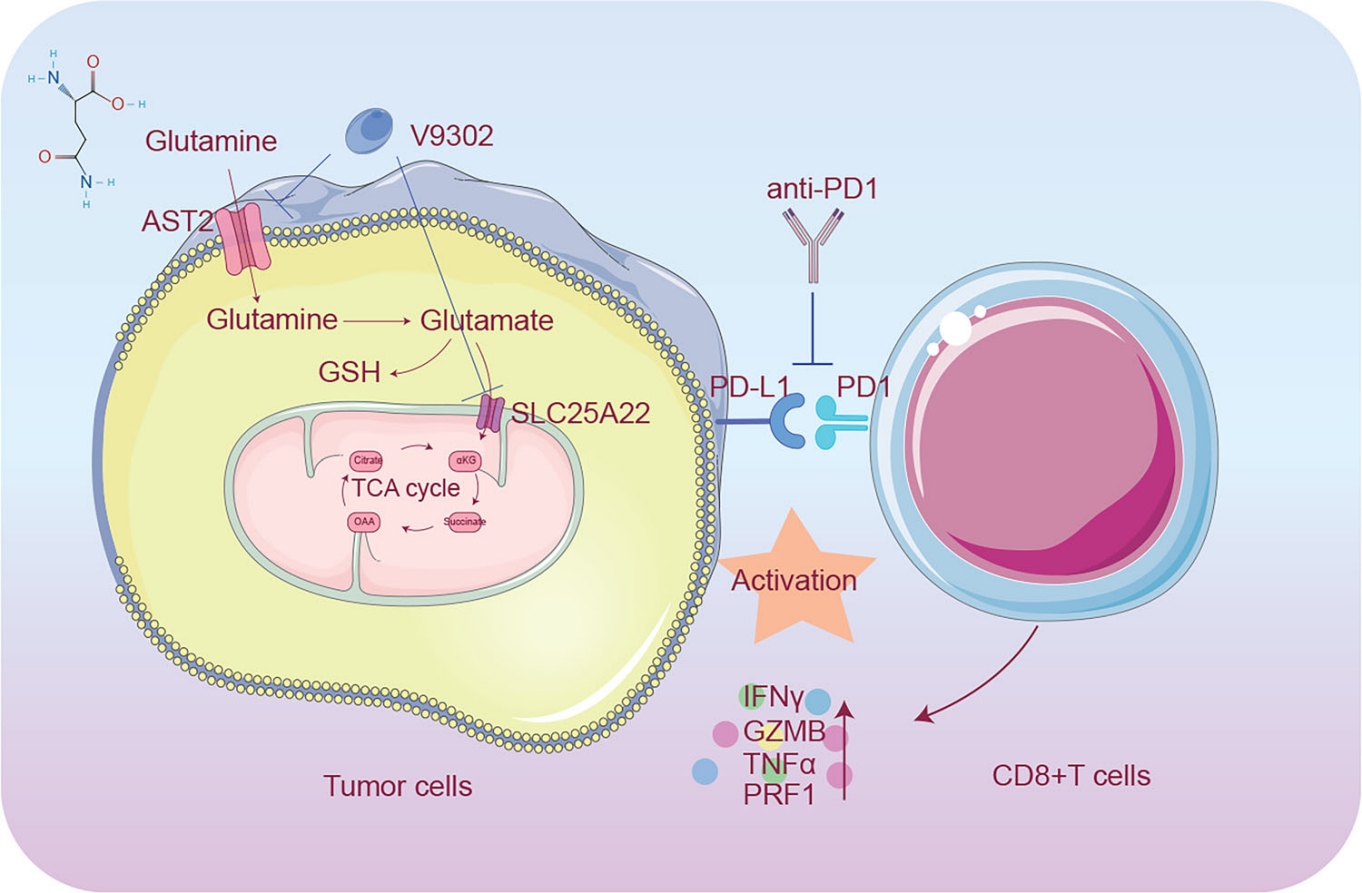
The possible mechanism of V9302 sensitizing anti‐PD‐1 therapy. Schematic representation of the proposed mechanism illustrating the potential mechanisms underlying the sensitization of PD‐1 through V9302. The administration of V9302 targeting SLC25A22 leads to enhanced secretion of cytotoxic effectors of CD8+T cells, augmenting sensitivity to anti‐PD‐1 therapy.

In summary (**Figures** **9** and **10**), 1) by performing metabolomic and transcriptomic sequencing, we identified a notably glutamine‐enriched/immunosuppressive microenvironment in CSCC. 2) By integrating metabolomic and transcriptomic analyses, we revealed that the glutamine transporter SLC25A22 potentially contributes to the development of an immunosuppressive microenvironment and acts as a crucial mediator linking glutamine metabolism, immune checkpoint activation, and the cytotoxicity of CD8+ T cells in CSCC. 3) Additionally, clinical validation using IHC, mIHC, and flow cytometry in CSCC samples demonstrated not only a concurrent increase in SLC25A22 and PD‐1 expression, coupled with reduced CD8+ T‐cell cytotoxicity but also revealed that SLC25A22 was overexpressed in CSCC patients with high PD‐L1 expression, suggesting that targeting SLC25A22 may improve the therapeutic efficacy of anti‐PD‐1 therapy, particularly in high PD‐L1 expression patients. 4) Furthermore, to explore the therapeutic value of targeting SLC25A22, we constructed 3D CSCC organoids that retained the morphological and genomic features of parental tumors and developed an innovative 3D T‐cell‐incorporated CSCC organoid models mimicking the interaction between tumor cells and T cells. Using this model, we demonstrated the crucial role of SLC25A22 in the malignant behavior of CSCC and that targeting the glutamine transporter SLC25A22 via V‐9302 facilitated the enhancement of CD8+ T‐cell cytotoxicity and increased the efficacy of anti‐PD‐1 therapy. 5) Moreover, our results from an in vivo xenograft model further supported the notion that targeting SLC25A22 via V‐9302 could effectively enhance the response to anti‐PD‐1 therapy. These discoveries provide new avenues for future exploration of strategies for increasing patient sensitivity to anti‐PD‐1 therapy through targeted metabolic intervention, laying a foundation for upcoming clinical trials.

## 3 Discussion

Although immunotherapy targeting immune checkpoints such as PD‐L1/PD‐1 and CTLA4 has shown promising results in enhancing the immune response against CSCC, the overall response rates remain relatively modest, ranging from 4% to 26%.^[^ ^26^, ^27^, ^28^ ^]^ This limited efficacy may be attributed to the TIME, which plays a critical role in tumor progression and significantly affects treatment outcomes.^[^ ^29^, ^30^ ^]^ In addition, extensive studies suggest that metabolism is a key determinant of antitumor immune response within the TIME,^[^ ^31^, ^32^ ^]^ yet its role in CSCC remains largely unexplored. To address this gap, 1) we conducted comprehensive metabolomic and transcriptomic analysis of 44 CSCC and 18 normal tissues, revealing a glutamine‐enriched /immunosuppressive microenvironment in CSCC. 2) Additionally, integrative metabolomic and transcriptomic analysis revealed that the glutamine metabolism transporter SLC25A22 contributed to the development of an immunosuppressive microenvironment and acted as a critical mediator linking glutamine metabolism, immune checkpoint activation, and the cytotoxicity of CD8+ T cells. 3) Furthermore, by IHC, mIHC, and flow cytometry validation in clinical CSCC samples, we not only demonstrated a simultaneous increase in SLC25A22 and PD‐1 expression accompanied by a reduction in CD8+ T‐cell cytotoxicity but also observed elevated SLC25A22 expression in CSCC patients with high PD‐L1 expression, which suggested that targeting SLC25A22 may enhance the cytotoxicity of CD8+ T cells and the efficacy of anti‐PD‐1 therapy, particularly in patients with high PD‐L1 expression. 4) To explore the therapeutic value of targeting SLC25A22 in CSCC, we initially developed a novel 3D CSCC organoid that accurately recapitulates the histopathological and genetic features of parental tumors and further constructed a 3D T‐cell‐incorporated CSCC organoid model that could mimic the interaction between CD8+ T cells and tumor cells. Moreover, we demonstrated the crucial role of SLC25A22 in the malignant behavior of CSCC and demonstrated that targeting the glutamine transporter SLC25A22 with V‐9302 enhanced the cytotoxicity of CD8+ T cells and promoted the effectiveness of anti‐PD‐1 therapy with an in vitro 3D T‐cell‐incorporated CSCC organoid model. 5) Further in vivo xenograft model experiments reaffirmed that V‐9302 was effective at enhancing the response to anti‐PD‐1 therapy. Collectively, these findings not only deepen our understanding of the complex interplay between high glutamine metabolism and the immunosuppressive microenvironment but also provide valuable guidance for future clinical trials on modulating glutamine metabolism to enhance the immunotherapy response in CSCC patients.

Metabolic alterations in the TIME can regulate immunity in cancer patients.^[^ ^33^ ^]^ Recent studies have highlighted the intricate interplay between metabolism, tumor cells, and immune cells in shaping tumor growth and antitumor immunity.^[^ ^34^ ^]^ However, the role of metabolism in the TIME and its functionality in antitumor immunity in CSCC remain largely unknown. In this study, our metabolomic analysis of 44 CSCC and 18 normal samples revealed elevated levels of glutamine metabolism in CSCC tissues, which was corroborated by metabolic sequencing of CSCC cell lines. Concurrent transcriptional analysis revealed an immune‐inhibitory microenvironment in CSCC characterized by infiltration of immunosuppressive cells and upregulation of the PD‐1 checkpoint pathway. Excitingly, by integrating metabolic and transcriptional data, we discovered the glutamine metabolism transporter SLC25A22 as a key mediator connecting glutamine metabolism, immune checkpoint activation, and CD8+ T‐cell cytotoxicity. Subsequent clinical validation further confirmed not only a simultaneous increase in SLC25A22 and PD‐1 expression accompanied by a reduction in CD8+ T‐cell cytotoxicity but also increased SLC25A22 levels in CSCC patients with high PD‐L1 expression, suggesting that targeting the metabolism transporter SLC25A22 may enhance CD8+ T‐cell cytotoxicity, reduce PD‐1 expression, and ultimately improve the efficacy of anti‐PD‐1 therapy, especially in CSCC patients with high PD‐L1 expression.

Recently, glutamine has been exploited as a “metabolic checkpoint” with metabolic plasticity between cancer cells and effector T cells, and several studies have shown that the glutamine transporter inhibitor V‐9302 can effectively inhibit glutamine uptake.^[^ ^35^, ^36^ ^]^ SLC25A22 has been previously implicated in tumorigenesis and immune modulation in colorectal cancer;^[^ ^24^, ^25^ ^]^ however, its role in CSCC remained unexplored. Our 2D CSCC cell culture experiments revealed that knockdown of SLC25A22 inhibited the malignant behavior of CSCC cells. To further explore the therapeutic implications especially immune response of targeting SLC25A22, we employed the glutamine transporter inhibitor V‐9302 in combination with PD‐1 blockade. However, 2D tumor cell lines cannot reflect the heterogeneity and diversity of tumors derived from patients and that the addition of immune cells to 2D cell cultures cannot restore the interaction patterns between tumor cells and immune cells in the TIME.^[^ ^37^ ^]^ Therefore, it is crucial to construct models that can simulate the in vivo tumor immune microenvironment. To address this issue, we developed CSCC organoids and performed histological characterization, transcriptional and genomic profiling to confirm the biologically consistentance with the original tissues. Building upon these findings, we further developed 3D T‐cell‐incorporated CSCC organoids that accurately portrayed the dynamic relationship between tumor cells and T cells. In summary, we constructed a research framework using 3D T‐cell‐incorporated CSCC organoids as an effective functional tool, providing model support for subsequent preclinical exploration of the effects of immunotherapy, such as anti‐PD‐1 therapy, on the glutamine metabolism transporter SLC25A22.

Given the inhibitory effect of the glutamine metabolism transporter inhibitor V‐9302 on glutamine metabolism^[^ ^36^ ^]^ and the successful establishment of 3D T‐cell‐incorporated CSCC organoids, we conducted preclinical exploration through in vitro and in vivo experiments using V‐9302. The results revealed that in the 3D T‐cell‐incorporated CSCC organoid model, both V‐9302 and PD‐1 blockers increased the production of proinflammatory cytokines (IFN‐γ and TNF‐α), which is crucial for the effectiveness of the immune response and cytotoxic molecules (CD107a, GZMA, GZMB, and PRF1) in CD8+ T cells, which is consistent with previous studies that have shown the importance of glutamine metabolism in T‐cell function and antitumor immune response.^[^ ^25^, ^38^, ^39^ ^]^ Importantly, through organoid‐T cell coculture and murine models, we show that targeting SLC25A22 enhances PD‐1 immunotherapy efficacy by downregulating immune checkpoints and boosting CD8+ T cell effector molecule secretion. This contrasts with its role in KRAS‐mutant CRC, where SLC25A22 promotes MDSC recruitment via asparagine‐SRC‐CXCL1 signaling.^[^ ^25^ ^]^ This dichotomy underscores the importance of tailoring strategies to tumor‐specific immune landscapes. Further studies should explore whether SLC25A22‐linked metabolic rewiring universally impacts checkpoint expression in epithelial cancers and validate its therapeutic potential in models recapitulating human immune microenvironment heterogeneity.

In conclusion, via metabolomic and transcriptomic analyses, we revealed a glutamine metabolism‐enriched, immunosuppressive TIME in CSCC. Integrated multi‐omics analysis identified SLC25A22 as a key link between glutamine metabolism, immune checkpoint activation, and CD8+ T‐cell cytotoxicity. Moreover, clinical sample analysis (IHC, mIHC, and flow cytometry) confirmed the co‐upregulation of SLC25A22 and PD‐1, the reduction of CD8+ T‐cell cytotoxicity, and the positive association between SLC25A22 and PD‐L1 expression, suggesting that targeting SLC25A22 may enhance anti‐PD‐1 efficacy, particularly in PD‐L1‐high patients. To validate this, we developed 3D CSCC organoid models and T‐cell‐incorporated organoid systems that mimic tumor‐immune interactions. Using these models, we demonstrated that V‐9302, a glutamine transporter inhibitor targeting SLC25A22, enhances CD8+ T‐cell cytotoxicity and anti‐PD‐1 effectiveness. This finding was further validated in an in vivo xenograft model.

Overall, our study provides novel insights into the metabolic and immune landscape of CSCC, highlighting SLC25A22 as a potential target to improve immunotherapy sensitivity and offering new directions for metabolism‐based therapeutic strategies.

## 4 Experimental Section

### Patient Samples and Cell Lines

CSCC and normal cervical tissue samples, along with peripheral venous blood, were obtained from the Tissue Bank of Obstetrics and Gynecology Hospital, Fudan University. Primary tumor and normal tissues were flash‐frozen and subsequently used for metabolite profiling, RNA extraction, and sequencing. Each tumor specimen was divided into two parts: one for RNA extraction and the other for metabolite analysis. All patient samples were de‐identified and anonymized. This study was approved by the Ethics Committee of Obstetrics and Gynecology Hospital of Fudan University, and written informed consent was obtained from all patients (2022‐129). Detailed clinical characteristics of these samples are provided in Table S1 (Supporting Information).

Siha and Hela cell lines were cultured in Dulbecco's Modified Eagle's Medium (DMEM) (Gibco 11 965 118) supplemented with 10% fetal bovine serum (FBS) and 1% penicillin‐streptomycin. All cell lines were sourced from ATCC and used for experiments within 10–20 passages post‐thawing. Authentication was confirmed via short tandem repeat (STR) testing. To ensure the absence of mycoplasma contamination, routine screenings were conducted using the MycoAlert Detection Kit (Lonza, LT07‐218).

### T Cell‐Incorporated CSCC Organoids

Organoids without T cells were generated from mixed tumor cells within one week to preserve the cellular composition of CSCC tumors. Peripheral blood mononuclear cells (PBMCs) were isolated from patients' peripheral blood as previously described. The PBMC culture medium (“T cell medium”) consisted of RPMI 1640 (Gibco), supplemented with 2 mm Ultraglutamine I, 1:100 penicillin/streptomycin, and 10% male human AB serum (Sigma‐Aldrich). One day before co‐culture, PBMCs were thawed in pre‐warmed (37 °C) T cell medium, with human serum replaced by fetal calf serum (FCS) during thawing. The cells were then incubated for 15 min with 25 U mL^−1^ benzonase (Merck), washed, and resuspended at a concentration of 2–3 × 10⁶ cells mL^−1^ in T cell medium supplemented with 150 U mL^−1^ IL‐2. PBMCs were cultured overnight at 37 °C before co‐culture. To prepare for co‐culture, tumor organoids were stimulated overnight with 200 ng mL^−1^ human recombinant IFN‐γ (Peprotech). The organoids were then combined with anti‐CD3/anti‐CD28‐activated PBMCs at a ratio of 1:100 in cold culture medium containing 3% Matrigel and incubated in a pre‐warmed (37 °C) six‐well microplate with an ultra‐low attachment surface for 36 h. This process facilitated the generation of T cell‐incorporated tumor organoids, in which PBMCs aggregated within the Matrigel surrounding the CSCC organoids and infiltrated into the tumor structures. T cell‐incorporated tumor organoids with diameters ranging from 100 to 300 µm were selected for cytotoxicity assays and drug screening experiments.

### Cell Viability Measurement

Cell viability was assessed using the CCK‐8 assay in CSCC cell lines. Cells were seeded in 96‐well plates at a density of 1 × 10⁴ cells per well and incubated for 24 h before treatment. The culture medium was then replaced with 100 µL of FBS‐supplemented medium containing various concentrations of free V‐9302 or V‐9302 in combination with other treatments, followed by incubation for 48 h. After treatment, the medium was removed, and 100 µL of serum‐free medium containing 10 µL of CCK‐8 solution (5 mg mL^−1^) was added to each well. Following a 4 h incubation, the absorbance at 450 nm was detected and the relative cell viability (%) was calculated as 100% × (Asample‐Ablank)/(A control‐Ablank).

### Immunohistochemistry

Immunohistochemistry was performed as described in the previous study.^[^ ^39^ ^]^ Briefly, tumor tissue slides were rehydrated, subjected to antigen retrieval, blocked, and incubated with primary antibodies. After a 6 h incubation, slides were treated with secondary antibodies and developed using the DAB peroxidase substrate kit for IHC (Yeasen, China). Three representative areas from each patient tissue sample were photographed at 200× magnification, and the density of positive staining was quantified using integrated absorbance and image area analysis. According to the percentage of positive cells, the IHC staining area score was divided as follows: 0 point (<5%),1 point (5%‐25%), 2 points (26%‐50%), 3 points (51%‐75%), and 4 points (76%–100%), whereas the IHC staining intensity was divided as follows: 0 point (negative); 1 point (weak positive); 2 points (positive); 3 points (strong positive). Immunoreactive scores were calculated by the multiplying the area and intensity scores. A list of the primary and secondary antibodies used in this study is provided in Table S2 (Supporting Information).

### Immunofluorescence Staining

Samples were collected onto glass slides and fixed with 4% paraformaldehyde (Boster, Wuhan, China) for 15 min. After rehydration, antigen retrieval, and blocking, the samples were incubated with primary antibodies diluted in blocking solution under parafilm at 4 °C overnight. Following washes in PBT (0.05% Tween in PBS), sections were incubated with fluorochrome‐conjugated secondary antibodies and counterstained with DAPI (1 µg mL^−1^, Sigma–Aldrich, MO, USA) for 5 min. Coverslips were then air‐dried and mounted. Images were acquired using a Zeiss LSM880 confocal microscope and processed with Zen software and ImageJ software. A list of the primary and secondary antibodies used in this study is provided in Table S2 (Supporting Information).

### Flow Cytometry

To detect intracellular proteins, cells were stimulated for 6 h with 10 µg mL^−1^ brefeldin A and 2 µm of ionomycin (Absin) before staining. Then, the cells were fixed and permeabilized using a fixation/permeabilization wash buffer (BioLegend) and stained with antibodies for 30 min in the dark. Intracellular staining for GZMB, PRF1, IFN‐γ, and TNF‐α was accomplished using a Cytofix/Cytoperm solution kit (BD, 554 714) on paraformaldehyde‐fixed cells, per the manufacturer's directions. Dead cells were excluded from analysis by staining with Ghost Dye Violet 450 (Tonbo, 13–0863) or Violet 510, as indicated. All the antibodies used in this study are listed in Table S2 (Supporting Information). All samples were run on a CytoFLEX platform (Beckman Coulter) and analyzed using FlowJo version 10.8 software (BD Biosciences). Flow cytometry gating strategy for identifying CD8+ T cells and cytotoxic effectors CD8+T cells were shown in Figure S6 (Supporting Information).

### Metabolite Extraction

The flash frozen patient tumors and normal cervical tissues were first cut in small pieces on dry ice, then 30 to 100 mg sections were weighed in a new 2 mL Eppendorf tube. The tissue samples with 200 µL of H2O and five ceramic beads were homogenized using the homogenizer. Eight hundred microliters methanol/acetonitrile (1:1, v/v) were added to homogenized solution for metabolite extraction. The mixture was centrifuged for 20 min (14 000 g, 4 °C). The supernatant was dried in a vacuum centrifuge. For LC‐MS analysis, the samples were re‐dissolved in 100 µL acetonitrile/water (1:1, v/v) solvent and centrifuged at 14 000 g at 4 °C for 15 min, then the supernatant was injected. To monitor the stability and repeatability of instrument analysis, quality control (QC) samples were prepared by pooling 10 µL of each sample and analyzed together with the other samples. The QC samples were inserted regularly and analyzed in every 5 samples.

### Metabolomic Analysis

In this study, a newly developed precision‐targeted metabolomics method^[^ ^40^ ^]^ was adopted to analyze the metabolomes of interest from trial samples (tissues and cell supernatant). Briefly, The metabolomics method was developed with a UPLC system (1290 Infinity series, Agilent Technologies) coupled to a triple quadrupole mass spectrometer (Agilent 6495 Series Triple Quad LC‐MS System). An ACQUITY UPLC HSS T3 column (2.1 mm i.d. × 100 mm, 1.8 µm; Waters) was used to analyse the metabolomes of interest. The samples were placed in an autosampler maintained at 10 °C, and then 5 µL were injected for LC‐MS analysis.

### Metabolomics Data Analysis

The collected LC‐MS raw data files were processed with quantitative analysis software (Agilent MassHunter Workstation System) for automatic peak recognition of the metabolites. To guarantee the high quality of metabolome data, the peak signals of metabolites with low abundance (<10^3^) were manually excluded during the data outlook The annotated metabolites from Kyoto Encyclopedia of Genes and Genomes (KEGG) Compound database (www.genome.jp/kegg) ^[^ ^41^ ^]^ and human metabolome database (HMDB; www.hmdb.ca)^[^ ^42^ ^]^ were used for further analysis. Next, the data file was used for analysis and visualization, including supervised discriminant analysis, volcano plots, and unsupervised heatmap combined hierarchical cluster analysis (HCA). MS data were normalized with the total sum of all detected ions, centered and scaled using Pareto scaling, which divided each variable by the square root of the standard deviation to suppress noise interference. An unpaired t‐test was used for the comparisons between the CSCC and NC groups to identify significantly differential metabolites (*p* < 0.05).

### Differential Abundance (DA) Score

DA score captures the tendency for a pathway to had increased levels of metabolites, relative to a control group. Then, after determining which metabolites were significantly increased/decreased in abundance, the DA score was defined as: DA (No. of metabolites increased − No. of metabolites decreased)/ No. of measured metabolites in pathway. The detected metabolites were first assigned to different metabolic pathway categories based on the KEGG pathways by MetaboAnalyst 5.0 software.^[^ ^43^ ^]^ Then SAM analysis method was applied to determine which metabolites are significantly increased or decreased in abundance in a pathway between different sample groups. Then, after determining the significant increased/decreased metabolites, the DA score was calculated. Thus, the DA score varies from −1 to 1. A score of 1 indicates that all metabolites in a pathway increased in abundance, while a score of −1 indicates that all metabolites decreased. Consider a limited detection capability by LC‐MS but important role of metabolites for some pathways. Pathways with no less than 2 detected metabolites were used for DA score calculation.

### Transwell Migration/Invasion Assay

The transwell invasion assay was performed using Matrigel diluted at a 1:50 ratio. CSCC cells were seeded in the upper chamber containing serum‐free DMEM, while the lower chamber was filled with DMEM supplemented with 10% FBS. After 48 h of incubation, the invaded cells in the lower chamber were stained with crystal violet and photographed. The cell migration assay followed the same procedure but was conducted without the addition of Matrigel.

### Wound‐Healing Migration Assay

For the wound‐healing migration assay, a scratch was made in the cell monolayer using a 200 µL pipette tip when the cell confluence reached ≈80%. Detached cells were then washed and discarded. Representative images were captured under a microscope at 0, 24, 48, and 72 h post‐injury. Each experiment was performed in triplicate.

### Cell Transfection

The SLC25A22‐knockdown, SLC25A22‐overexpression, and control lentiviruses were constructed by Shanghai Genomeditech Co., Ltd. MS751 cells were digested from the dish in logarithmic growth, resuspended, and seeded in petri dish at 5 × 10^5^ cells per well, and cultured overnight. Transduction was performed when the cells reached 80% confluence. Briefly, 2 µg of the virus stock was diluted in 200 µl of serum‐free DMEM in a 1.5 ml EP tube, and the virus solution was incubated at room temperature for 15–20 min. The diluted virus was evenly added to the cells in fresh medium, drop by drop, after 24 h, check the transduction efficiency, and continue the experiments.

### Metabolomics and Stable‐Isotope Tracing

Cancer cell lines were maintained in DMEM supplemented with 10% FBS at 37 °C in a 5% CO2 incubator. Following a 2 h starvation period in glutamine‐free medium, cells were exposed to 2 mm ¹^3^C‐labeled glutamine for 24 h. After incubation, cells were washed with ice‐cold PBS, and metabolites were extracted using 80% methanol. The resulting supernatant was stored at −80 °C until analysis. Isotopic incorporation into key metabolites was assessed using GC‐MS or LC‐MS.

### Transcriptome Examination and Analysis

Total RNA was isolated from tumors and normal cervical tissues using TRIzol Reagent according to the manufacturer's instructions. mRNAseq libraries were made from total RNA using the Illumina TruSeq mRNA sample preparation kit and sequenced on an Illumina HiSeq 2500 using a 2 × 50 bp configuration. All the assembled transcripts were searched against the national center for biotechnology information (NCBI) protein nonredundant (NR), string, and Kyoto Encyclopedia of Genes and Genomes (KEGG) databases using BLASTX. BLAST2GO program was used to get Gene Ontology (GO) annotations of unique assembled transcripts for describing biological processes, molecular functions, and cellular components. To identify differential expression genes among different treatments, the expression level of each transcript was calculated according to the fragments per kilobase of exon model per million mapped reads. RSEM was used to quantify gene and isoform abundances. In addition, functional‐enrichment analyses including GO and KEGG were performed to identify which differential expression genes were significantly enriched in GO terms and metabolic pathways at Bonferroni‐corrected *p* value ≤ 0.05 compared to the whole‐transcriptome background.

### In Vivo Murine Tumor models

All experiments were performed under the approval of Fudan University Animal Care and Use Committee (202312011S). Female C57BL/6 mice (6–8 weeks old) were purchased from the Laboratory Animal Center of the Shanghai Institutes for Biological Sciences and were housed in a pathogen‐free environment. To establish ectopic tumors, 5 × 10^5^ TC‐1 cells (in 100 µl of PBS) were injected subcutaneously into the right shoulders of C57BL/6 mice. When the tumor size reached ≈50 mm^3^, the tumor‐bearing mice were randomly divided into four groups (n = 6 for each group): the control, anti‐PD1, anti‐V9302, and anti‐PD1‐V9302 groups (denoted as Day 0). Tumor‐bearing mice in the treatment group were received intravenous injection of 0.2 mg of PD‐1 mAb or immunoglobulin G control mAb in 100 µL of PBS from day 8 for a total of 4 doses in the absence or presence of vehicle (DMSO) or V‐9302 (30 µm) for 48 h. For immune response analysis, the tumors were excised on day 21. Tumor tissues were digested in the RPMI 1640 medium supplemented with 0.2% collagenase type IV, 0.01% hyaluronidase, and 0.002% DNase I for 3 h at 37 °C in 5% carbon dioxide. The TILs were separated using Ficoll (Dakewe Biotech Company), followed by flow cytometry analysis. Tumor size was measured by caliper and body weight was recorded every 3 or 4 days. Tumor volumes were calculated with the formula: volume = (L×W×W)/2, where L is the tumor length and W is the tumor width measured in millimeters. All mice were observed for overall condition and weighed weekly. For the survival study, mice were sacrificed when tumor reached 2 cm in any dimension.

### Gene/Metabolite Expression Data Set Analysis

To identify differentially expressed genes/metabolites (DEGs/DEMs) among groups, the function FindMarkers with Wilcoxon rank sum test algorithm was used. Significantly DEGs were selected as those meeting the following criteria: 1) log(fold change) > 0.25, 2) p‐value < 0.05, and 3) min.pct > 0.1. GO analysis was performed to elucidate the biological implications of DEGs/DEMs and marker genes.^[^ ^44^ ^]^ GO annotations were downloaded from the NCBI (http://www.ncbi.nlm.nih.gov/), GO (http://www.geneontology.org/), and UniProt (http://www.UniProt.org/) databases. The Fisher exact test was used to identify the significant GO categories, and a false discovery rate (FDR) was used to correct the p‐values. Pathway analysis was used to explore the significant pathways of the DEGs/DEMs and marker genes based on the KEGG database. The Fisher exact test was used to identify significant pathways, and the threshold of significance was defined using the p‐value and false discovery rate (FDR).

### Statistical Analysis

All statistical analysis was conducted using Prism 8.0 (GraphPad Software). All graphs depict mean ± SEM unless otherwise indicated. Statistical significances were denoted as not significant (ns; *P* > 0.05), *p < 0.05, **p < 0.01, ***p < 0.001, ****p < 0.0001. The numbers of experiments were noted in figure legends. To assess the statistical significance of single metabolites between two groups, Mann‐Whitney test, Welch's t test, and unpaired T‐test were used. For animal experiments comparing more than two conditions, differences were tested by a one‐way ANOVA followed by Dunnett's or Tukey's multiple comparison tests.

## Conflict of Interest

The authors declare no conflict of interest.

## Author Contributions

T.R., J.Q., F.C., and Q.J. contributed equally to this work. H.J. and K.H. initiated the project and designed and supervised the research plan. T.R., Q.L., and Q.J. T.W. performed stimulation experiments under the supervision of T.R., F.C., and J.Q. wrote the manuscript. T.R. and Q.J. designed and performed the supplementary experiments. Q.J., K.H., H.J., and J.Q. made significant revisions in language to the manuscript. The order of first authorship was determined by contribution to project design. All authors edited and approved the manuscript. K.H.  is responsible for the overall content as guarantor.

## Supporting information



Supporting Information

## Data Availability

The data that support the findings of this study are available from the corresponding author upon reasonable request.
