# Supplementary material for: Targeting Glutamine Metabolism Transporter SLC25A22 Enhances CD8+ T‐Cell Function and Anti‐PD‐1 Therapy Efficacy in Cervical Squamous Cell Carcinoma: Integrated Metabolomics, Transcriptomics and T‐Cell‐Incorporated Tumor Organoid Studies
Source: Adv Sci (Weinh). 2025 Jun 27;12(33):e02225. doi: 10.1002/advs.202502225 (PMC12412565; doi:10.1002/advs.202502225)
Supplement: Supplementary file 1 — Supporting Information [file ADVS-12-e02225-s001.docx]

**Supplementary materials**

**Supplementary figures:**


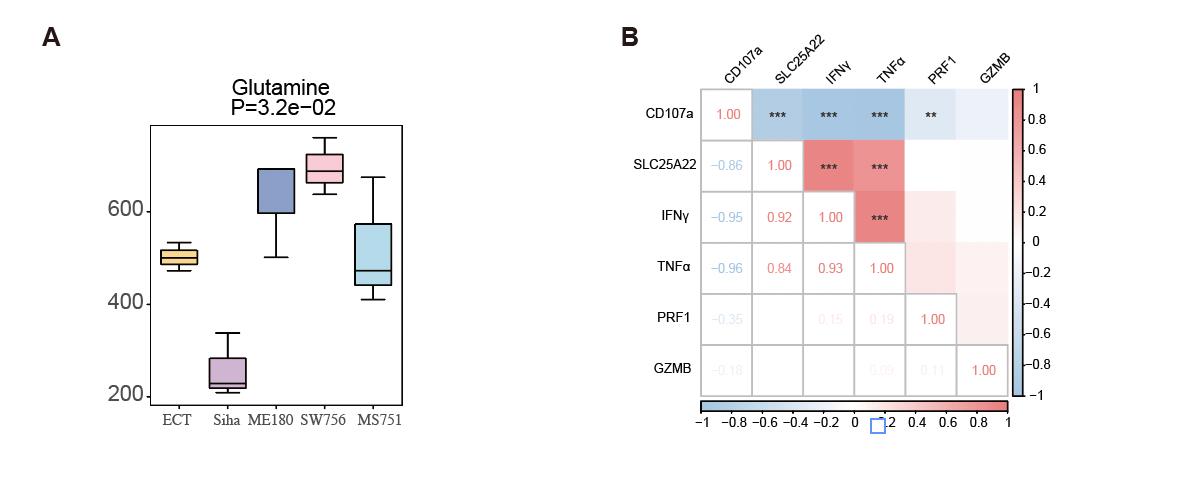


Fig. S1:(A)The barplot of expression level of glutamine among among CSCC cell lines (Siha, ME180, SW756, MS751) and cervical normal cell line (ECT). (Kruskal-Wallis test)

1. Correlation heatmap of SLC25A22 with cytotoxic markers in CD8+ T cells.(Pearson correlation，**p<0.005,***p<0.001)

Abbreviations: CSCC, cervical squamous cell cancer


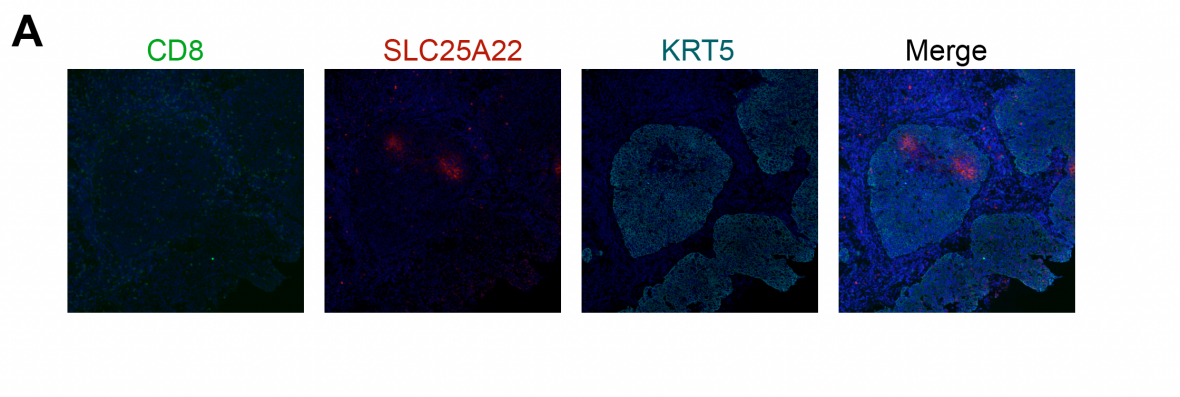


Fig S2: Representative immunofluorescence images showing tumor cells(KRT5+),CD8+T cells(CD8+) and the expression levels of SLC25A22.Original magnifications: ×100, scare bar:100μm.


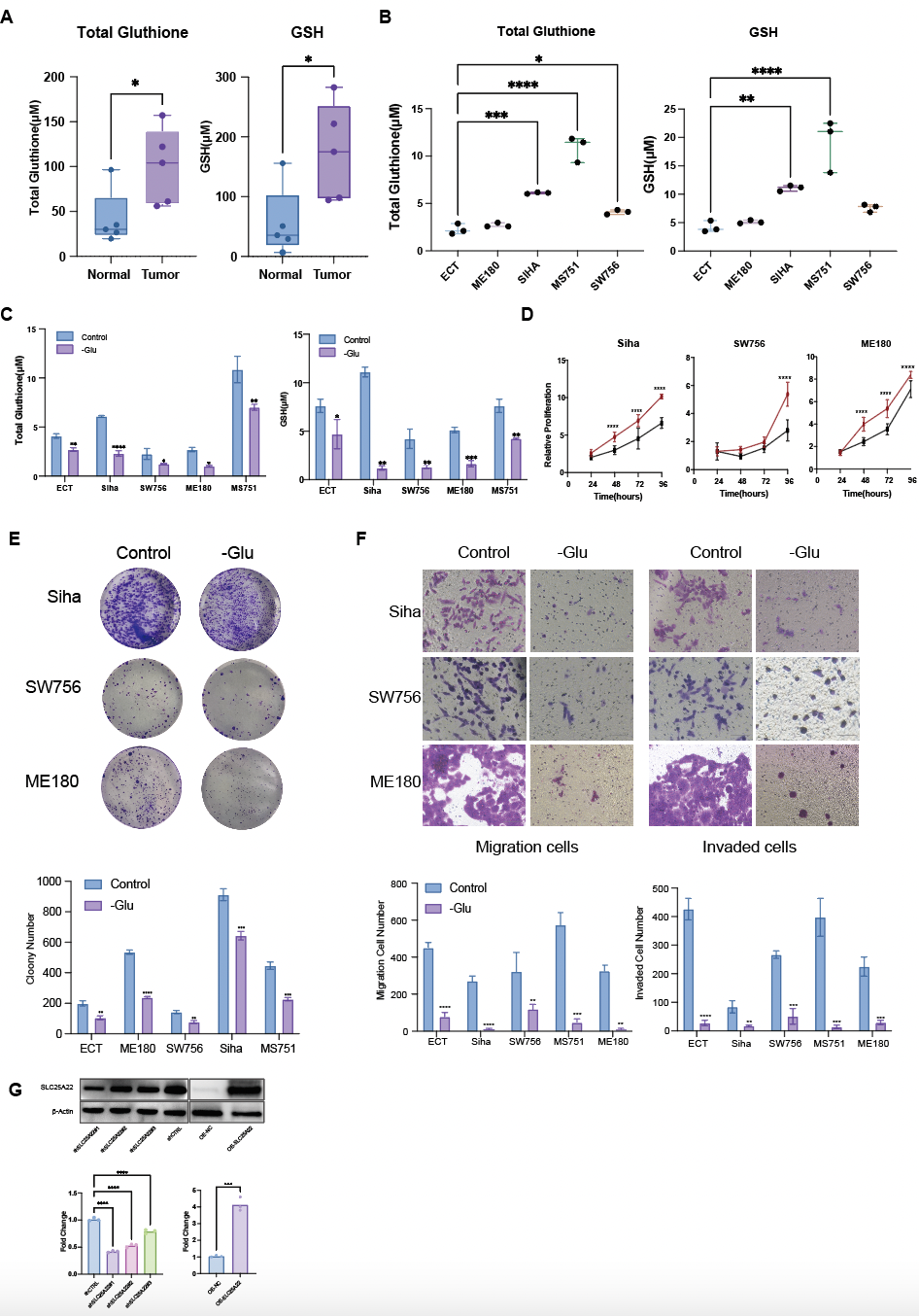


FigS3: Role of glutamine metabolism and SLC25A22 in the malignant progression of CSCC.

A barplot ahowed higher intensity of glutamine and glutathione in CSCC compared to normal tissues.(T-test, *p<0.05). (B) A barplot ahowed higher intensity of glutamine and glutathione in CSCC cell lines compared to normal cervical cell lines.(One-way ANOVA test, *p<0.05, ***p<0.001, ****p<0.0001). (C) A bar plot showed lower level of glutamine and glutathione under glutamine-deprived conditions in CSCC cell lines.(T-test, *p<0.05, ***p<0.001, ****p<0.0001). (D)CCK-8 assay showed a significant reduction of cell proliferation ability under glutamine-deprived conditions in CSCC cell lines (Siha/SW756/ME180). (Two-way ANOVA test, ****p<0.0001 )(E) Representive images (Up) and related statistical graphs (Down) of cell clone of Siha/SW756/ME180 cell lines.(T-test, **p<0.01, ***p<0.001, ****p<0.0001) (F) Representive images (Up) and related statistical graphs (Down) of trasnwell migration and invasion experiments of Siha/SW756/ME180 cell lines under glutamine-deprived conditions. Original magnifications: ×200, scare bar:100μm. (T-test,**p<0.01, ***p<0.001, ****p<0.0001 ) (G) Western blot of expression of SLC25A22 in Si-SLC25A22/Si-NC/OE-SLC25A22/OE-NC cell lines.(One-way ANOVA, ***p<0.001, ****p<0.0001)


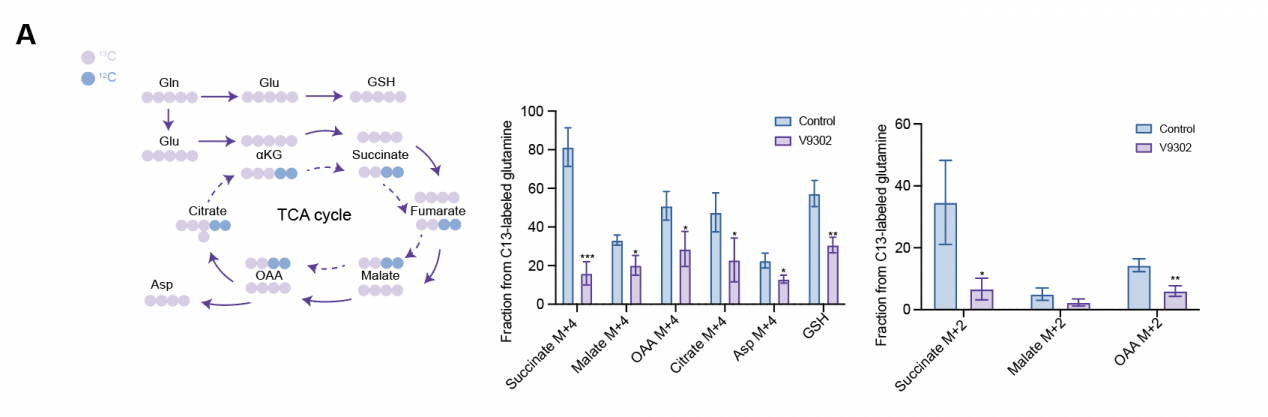


Fig. S4: V9302 inhibit the cell viability and proliferation ability of Siha (CSCC cell line) cells.

1. Metabolic flux analysis revealed that V9302 significantly impaired SLC25A22-mediated glutamine metabolism in the TCA cycle

CSCC, cervical squamous cell cancer;PBMC,peripheral blood mononuclear cells.


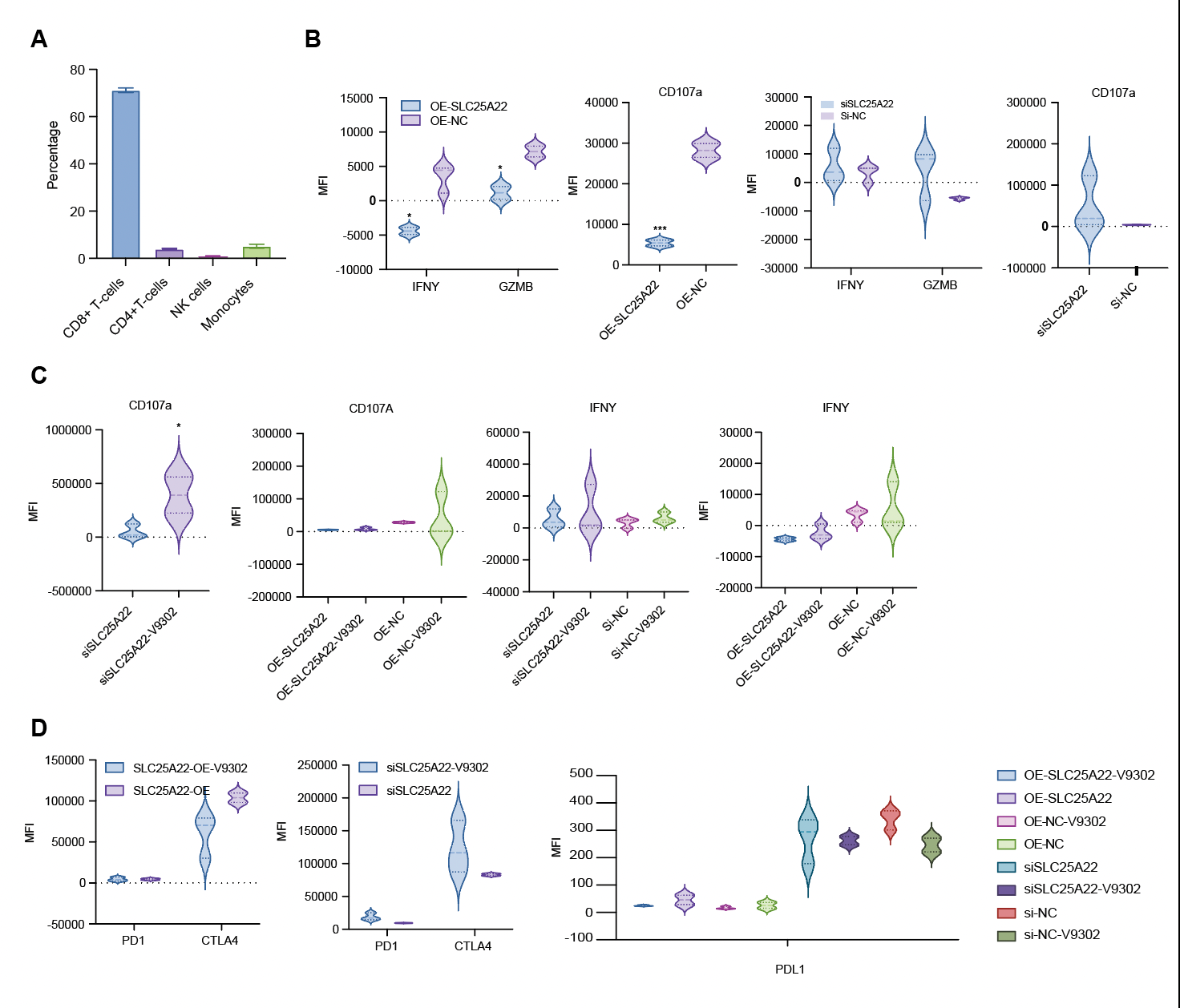


Fig S5: Targeting SLC25A22 using V9302 as promising therapy to sensitize anti-PD-1 therapy

1. A barplot showed the composition of immune cells in 3D T cell incorporated organoid. (B) Violin plot showed expression of effector molecules in CD8+ T cells co-cultured with SLC25A22 knockdown/overexpression cell lines. (T-test, Welch's t test, *p<0.05,***p<0.001)(C) Violin plot showed expression of effector molecules in CD8+ T cells co-cultured with SLC25A22 knockdown/overexpression cell lines under the treatment of V9302. (T-test,*p<0.05)(D) Violin plot showed MFI of PD1/PDL1/CTLA4 in CD8+ T cells co-cultured with SLC25A22 knockdown/overexpression cell lines under the treatment of V9302.


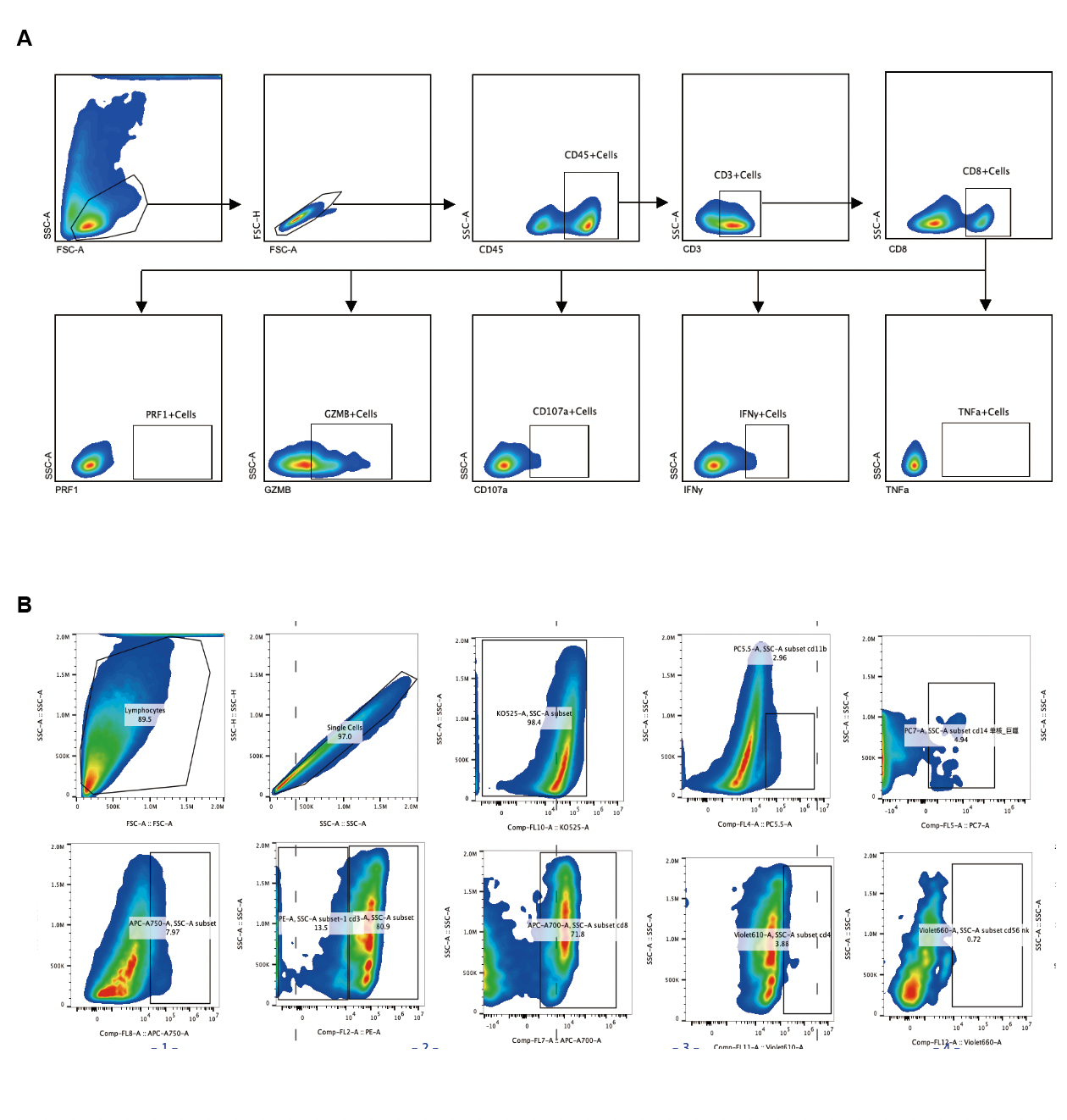


Fig. S6:Flow cytometry gating strategy

(A) Flow cytometry gating strategy for identifying immune cells in 3D T cell incorporated organoid. (B)Flow cytometry gating strategy for identifying CD8+ T cells and cytotoxic effectors CD8+T cells.

Live cells or peripheral blood mononuclear cells were first gated by the forward and side scatter areas, and doublets were then excluded by gating with the forward scatter area and height. Leukocytes were gated by CD45+ cells. Cells in the leukocyte gate were further gated based on CD3+CD8+ T cells. The positive gate of cytotoxic effectors were shown in the lower half of this figure.

**Supplementary tables:**

| **Patient ID** | **Age** | **HPV infection** | **FIGOA stage** | **Histological type** | **CPSB** |
| --- | --- | --- | --- | --- | --- |
| CC1 | 56 | yes | IIIC2 | SCCC | 9.049733333 |
| CC2 | 50 | yes | IIIC1 | SCC | N/A |
| CC3 | 59 | yes | IB2 | SCC | N/A |
| CC4 | 61 | yes | IIIA | SCC | N/A |
| CC5 | 56 | yes | IIIC1 | SCC | N/A |
| CC6 | 63 | yes | IIIC1 | SCC | N/A |
| CC7 | 67 | N/A | IB2 | SCC | N/A |
| CC8 | 59 | N/A | IIIC2 | SCC | N/A |
| CC9 | 53 | no | IB2 | SCC | 104.9443333 |
| CC10 | 59 | yes | IIIC1 | SCC | 1.2342 |
| CC11 | 25 | yes | IIIC1 | SCC | 9.853333333 |
| CC12 | 40 | yes | IIIC1 | SCC | 26.523 |
| CC13 | 73 | no | IIIC1 | SCC | 18.26873333 |
| CC14 | 69 | yes | IIIC1 | SCC | 102.854 |
| CC15 | 64 | yes | IB3 | SCC | N/A |
| CC16 | 58 | yes | IIIC1 | SCC | N/A |
| CC17 | 66 | yes | IB2 | SCC | 7.453 |
| CC18 | 67 | N/A | IIIC1 | SCC | 3.5072 |
| CC19 | 54 | yes | IB3 | SCC | 128.5733333 |
| CC20 | 59 | yes | IIB | SCC | 24.45233333 |
| CC21 | 67 | yes | IIIC2 | SCC | 83.88666667 |
| CC22 | 52 | N/A | IIB | SCC | 3.7644 |
| CC23 | 77 | yes | IIIC1 | SCC | 45.02733333 |
| CC24 | 46 | yes | IIIC1 | SCC | 46.77766667 |
| CC25 | 40 | yes | IB2 | SCC | 36.20913333 |
| CC26 | 46 | yes | IB3 | SCC | 0.4602 |
| CC27 | 71 | N/A | IIA2 | SCC | 2.0563 |
| CC28 | 31 | yes | IIA2 | SCC | 81.59133333 |
| CC29 | 54 | yes | IIIC1 | SCC | N/A |
| CC30 | 67 | yes | IIIC1 | SCC | N/A |
| CC31 | 48 | yes | IIA2 | SCC | 70.702 |
| CC32 | 47 | yes | IIA2 | SCC | N/A |
| CC33 | 62 | yes | IB2 | SCC | N/A |
| CC34 | 51 | no | IIIC1 | SCC | N/A |
| CC35 | 59 | yes | IB2 | SCC | N/A |
| CC36 | 62 | yes | IIIC2 | SCC | N/A |
| CC37 | 50 | yes | IB3 | SCC | N/A |
| CC38 | 51 | N/A | IIA2 | SCC | N/A |
| CC39 | 34 | yes | IIIC1 | SCC | N/A |
| CC40 | 43 | yes | IB3 | SCC | N/A |
| CC41 | 41 | yes | IB2 | SCC | N/A |
| CC42 | 45 | yes | IB2 | SCC | N/A |
| CC43 | 63 | yes | IIA1 | SCC | N/A |
| CC44 | 42 | yes | IIIC1 | SCC | N/A |
| AInternational Federation of Gynecology and Obstetrics BCombined Positive Score Csquamous cell carcinoma | | | | | |

Table S1: The baseline characteristics of 44 CSCC patients.

| Reagents and antibodies | | | |
| --- | --- | --- | --- |
| Type | Source | Cat No. | Name |
| Reagents | Selleck | S8818 | V-9302 |
|  | Bioxcell | BE0146 | invivo Mab anti-mouse PD-1 (CD279) |
|  | Bioxcell | BE0297 | InVivoMAb human IgG1 isotype control |
|  | BD Pharmingen | 564406 | Fixable Viability Stain 510 |
|  | BD Pharmingen | 564219 | Human BD Fc Block(Fc1.3216) |
| Antibodies | SIGMA | HPA014662-100UL | Anti-SLC25A22 antibody |
|  | Abcam | ab52635 | Anti-Cytokeratin 5 antibody [EP1601Y] - Cytoskeleton Marker |
|  | Abcam | ab150077 | Goat Anti-Rabbit IgG H&L (Alexa Fluor® 488) |
|  | Abcam | ab217344 | Anti-CD8 alpha antibody [EPR21769] |
|  | Abclonal | A0093 | Perforin Rabbit pAb |
|  | Abclonal | A12450 | IFN gamma Rabbit pAb |
|  | Abclonal | A24804 | LAMP1/CD107a Rabbit PolymAb® |
|  | Abclonal | A21265 | TNF-a Rabbit mAb |
|  | Abclonal | A2557 | Granzyme B Rabbit pAb |
|  | Abcam | ab150083 | Goat Anti-Rabbit IgG H&L (Alexa Fluor 647) preadsorbed |
|  | Cell Signaling Technology | 13684 | PD-L1 (E1L3N®) XP® Rabbit mAb |
|  | Cell Signaling Technology | 86163 | PD-1 (Intracellular Domain) (D4W2J) XP® Rabbit mAb |
|  | BD Pharmingen | 557833 | APC-Cy7 Mouse Anti-Human CD45(2D1) |
|  | BD Pharmingen | 555339 | FITC Mouse Anti-Human CD3(HIT3a) |
|  | BD Pharmingen | 561453 | Alexa Fluor 700 Mouse Anti-Human CD8(RPA-T8) |
|  | BD Pharmingen | 566558 | BB700 Mouse Anti-Human CD107a(H4A3) |
|  | BD Pharmingen | 560212 | Alexa Fluor 647 Mouse anti-Human Granzyme B(GB11) |
|  | BD Pharmingen | 563393 | BV421 Mouse Anti-Human Perforin(δG9) |
|  | BD Pharmingen | 563418 | BV650 Mouse Anti-Human TNF(MAb11) |
|  | BD Pharmingen | 557643 | PE-Cy7 Mouse Anti-Human IFN-γ(B27) |
|  | BD Pharmingen | 557659 | APC-Cy7 Rat Anti-Mouse CD45(30-F11) |
|  | BD Pharmingen | 563004 | BV605 Hamster Anti-Mouse CD3e(145-2C11) |
|  | BD Pharmingen | 553030 | FITC Rat Anti-Mouse CD8a(53-6.7) |
|  | BD Pharmingen | 562523 | PE-CF594 Hamster Anti-Mouse CD279 (PD-1)(J43) |
|  | BD Pharmingen | 567041 | R718 Rat Anti-Mouse TNF(MP6-XT22) |
|  | BD Pharmingen | 557649 | PE-Cy7 Rat Anti-Mouse IFN-γ(XMG1.2) |
|  | BD Pharmingen | 560647 | PE-Cy7 Rat Anti-Mouse CD107a(1D4B) |
|  | Thermo | 12-8898-82 | ANTI-MO GRANZYME B NGZB PE |
|  | Biolegend | 154316 | PE/Dazzle™ 594 anti-mouse Perforin Antibody |
|  |  |  |  |

Table S2: The reagents and antibodies used in this article.
